# The Role of *Aeromonas*-Goblet Cell Interactions in Melatonin-Mediated Improvements in Sleep Deprivation-Induced Colitis

**DOI:** 10.1155/2022/8133310

**Published:** 2022-03-20

**Authors:** Ting Gao, Zixu Wang, Jing Cao, Yulan Dong, Yaoxing Chen

**Affiliations:** ^1^College of Veterinary Medicine, China Agricultural University, Haidian, Beijing 100193, China; ^2^Department of Nutrition and Health, China Agricultural University, Haidian, Beijing 100193, China

## Abstract

**Background:**

Our previous studies demonstrated that melatonin could effectively ameliorate sleep deprivation- (SD-) caused oxidative stress-mediated gut microbiota disorder and colitis. The research further clarified the mechanism of melatonin in improving colitis from the perspective of the interaction between *Aeromonas* and goblet cells.

**Methods:**

A seventy-two hours SD mouse model with or without melatonin intervention and fecal microbiota transplantation (FMT) to explore the vital position of *Aeromonas*-goblet cell interactions in melatonin improving SD-induced colitis. Moreover, *Aeromonas* or LPS-supplied mice were assessed, and the influence of melatonin on *Aeromonas*-goblet cell interactions-mediated oxidative stress caused colitis. Furthermore, in vitro experiment investigated the regulation mechanism of melatonin.

**Results:**

Our study showed that SD induced colitis, with upregulation of *Aeromonas* and LPS levels and reductions in goblet cells number and MUC2 protein. Similarly, FMT from SD mice, *Aeromonas* veronii colonization, and LPS treatment restored the SD-like goblet cells number and MUC2 protein decrease and colitis. Moreover, LPS treatment downregulated the colonic antioxidant capacity. Yet, melatonin intervention reversed all consequence in SD, *A.*veronii colonization, and LPS-treated mice. In vitro, melatonin reversed *A.* veronii- or LPS-induced MUC2 depletion in mucus-secreting human HT-29 cells via increasing the expression level of Villin, Tff3, p-GSK-3*β*, *β*-catenin, and melatonin receptor 2 (MT2) and decreasing the level of p-I*κ*B, p-P65, ROS, TLR4, and MyD88 proteins, while the improvement effect was blocked with pretreatment with a MT2 antagonist but were mimicked by TLR4 and GSK-3*β* antagonists and ROS scavengers.

**Conclusions:**

Our results demonstrated that melatonin-mediated MT2 inhibits *Aeromonas*-goblet cell interactions to restore the level of MUC2 production via LPS/TLR4/MyD88/GSK-3*β*/ROS/NF-*κ*B loop, further improving colitis in SD mice.

## 1. Introduction

Sleep has a vital effect on the homeostasis of gastrointestinal mucosal barriers and intestinal microbiota [1, 2]. Insufficient sleep is closely related to lots of adverse outcomes, including higher risk of cardiovascular disease, diabetes mellitus, coronary heart disease, hypertension [3], and inflammatory bowel disease (IBD) [4]. In fact, some researches have indicated that sleep disorders may disrupt the immune homeostasis in the intestines, further inducing inflammatory response, and resulting in the occurrence of IBD [4]. Conversely, IBD patients also suffer from poor sleep quality and markedly prolonged sleep latency, as well as frequently sleep fragmentation [5], which highlights the closely correlation between IBD and sleep deficiency.

The intestinal homeostasis relies on closely regulated cross-talk between the mucosal immune, intestinal microbiota, and intestinal epithelial cells (IECs) [6]. Mucus layer damage accelerates intestinal epithelium-pathogen interactions and the pathogen invasion [7]. O-glycosylated mucin (MUC)2, produced by goblet cells, constitutes the major component of the intestinal mucus layer of the rodents' intestines and is a mechanical barrier by forming a huge network of mucus polymer barrier [8]. Importantly, the reduction of MUC2 content and goblet cells number means a thinner mucus layer, which is closely related to IBD [9]. Considering that mucin has a positive effect on offering protection resist the multiple inflammation caused by toxins and invading pathogenic bacteria, it is vital to distinguish the elements that regulate MUC2 gene expression exposed to insufficient sleep [10–12].

Melatonin (N-acetyl-5-methoxytryptamine, MT), synthesized from tryptophan, often used to regulate sleep. MT can regulate a series of molecular process, such as circadian rhythms, sleep control, immune pathway, oxidative stress, apoptosis, and autophagy [13]. Moreover, MT is a neurotransmitter among intestinal hormones, and it affects physiological functions of the gastrointestinal tract (GI), including bicarbonate secretion, motility, permeability, energy utilization, and tight junction proteins in intestines [14]. Specifically, consider that mucin influences the abundance of intestinal microbiota [15] and that some microbiota treats glycan as a nutrient [16, 17], bacteria modulation via melatonin may be due to goblet cells differentiation-mediated mucin regulation. Therefore, MT is expected to treat a variety of GI diseases, such as necrotizing enterocolitis, ischemic injuries, and IBD [13]. However, it remains unknown whether MT mediated the regulation of MUC2 synthesis and secretion improves IBD in response to SD. Thus, we aim to determine the influence of SD on MUC2 depletion and the roles of MT using a continuous 72 h SD mouse model and *Aeromonas* veronii or LPS-treated mucus-secreting human HT-29 cells.

## 2. Materials and Methods

All experiments were operated which subject to the Guide for the Care and Use of Laboratory Animals published by the Animal Welfare Committee of the Agricultural Research Organization, China Agricultural University (Approval No. CAU20170911-2).

### 2.1. Animal Model Establishment

168 male ICR mice (eight weeks old; Vital River Laboratory Animal Technology Co. Ltd., Beijing, China) were fed in 28 cages (six mice/cage) in general environments (temperature: 21 ± 1°C, relative humidity: 50 ± 10%) with a regular 10 h dark: 14 h light cycle (lights on at 7 : 00 am.). The mice eat and drink freely. One week acclimatization later, the mice were casually distributed to 14 groups and used to carry out four experiments: the sleep deprivation experiment: sleep deprivation (SD), SD + melatonin supplementation (SD + MT), and nonsleep-deprived control (CON) groups, the ZT time of day that SD started and MT administration were previously described by Gao et al. and Zhang et al.; [18, 19]; fecal microbiota transplantation (FMT) experiment: F-CON, F-SD, F − SD + MT (F-SM), and F-R group, FMT was performed via oral gavage of a feces into wild mice as described Stebegg et al.; [20] *Aeromonas* veronii colonization experiment: C-CON, C-*Aeromonas* (C-A), and C − *Aeromonas* + MT (C-AM) groups; and LPS experiment: LPS, LPS + melatonin supplementation (LPS + MT), LPS + TAK − 242 supplementation (LPS + TAK − 242), and non-LPS control (CON) groups.

Colitis was evaluated daily based on the overall rating of body weight, stool consistency, and fecal occult blood to count the disease activity index (DAI), which indicated in Table 1. The scoring range for DAI scores is 0-4, as previously recorded by Murthy et al. [21]. Specific study design is supplied in the Supplementary Material.

### 2.2. Fecal Occult Blood Test

Specific study design is supplied in the Supplementary Material. The judgment criterion is negative: (-) there is no rose red or cherry red after 3 minutes; positive: (+) rose red or cherry red appears within 30-60 s; strong positive: (++) rose red or cherry red appears immediately; and the strongest positive: (+++) a deep rose red or deep cherry red appears immediately.

### 2.3. Intestinal Permeability to Fluorescein Isothiocyanate- (FITC-) Dextran

Two hours before the end of the experiment (6 : 00 am), all mice were given oral administration of 0.6 mg/g body weight of 4-kDa FITC-dextran at a concentration of 80 mg/mL and fasted for 2 hours. Euthanasia was subsequently carried out. Blood was collected by retroorbital ocular hemorrhage and centrifuged (500 × *g*, 10 minutes) to collect serum. A fluorescence spectrophotometer with emission at 535 nm and excitation at 485 nm was used to calculate the fluorescence value in the serum. Create a standard curve via diluting FITC-dextran within PBS using a standard curve to calculate the content of FITC-dextran of the serum.

### 2.4. Histological Staining

Specific study design is supplied in the Supplementary Material. The scoring criteria are (I) 0: no obvious inflammation; (II) 2: low-grade inflammation, scattered infiltrating monocytes (1~2 lesions); (III) 4: multifocal lighter inflammation; (IV) 6: high level of inflammatory response and upregulated blood vessel density, as well as obvious thickening of the wall; and (V) 8: the most severe inflammation, accompanied by transmural leukocyte infiltration.

### 2.5. Immunohistochemical Staining

Using immunohistochemistry to stain for MUC2 in paraffin intestinal sections, tissues were infiltrated overnight at 4°C in the monoclonal rabbit anti-mouse primary antibody (MUC2, 1 : 500; TLR4, 1 : 200; Abcam, Cambridge, MA, USA). Specific operation is supplied in the Supplementary Material.

### 2.6. Enzyme-Linked Immunosorbent Assay (ELISA)

A competitive ELISA assay (Uscn Life Science, Inc., Wuhan, China) was used to assess the inflammatory factors (IL-10, TNF-*α*, IFN-*γ*, and IL-1*β*) and fecal LPS of colonic tissue. The operations were operated on the basis of the manufacturer's instructions. There were 8 samples in every group, and every sample was detected in triplicate. A microplate reader (Model 680, Bio-Rad, St. Louis, MO, USA) equipped with a 450 nm filter was used to calculate the data. The data were written as pg/mg protein for the IL-10, TNF-*α*, IFN-*γ*, and IL-1*β* levels of the colonic tissue and *μ*g/mL for the LPS of the colonic content.

### 2.7. PAS Staining

Colon tissues were soaked in 4% paraformaldehyde in 0.1 M phosphate-buffered saline (pH 7.4, 4°C) immediately for forty-eight hours and infiltrated in paraffin for sectioning (5 *μ*m, cross-section). All colonic tissues were embedded in periodic acid-schiff (PAS). 30 random fields in 6 sections of every sample from PAS staining were photographed at 400x magnification with a microscope (BX51; Olympus, Tokyo, Japan), and a total of at least 360 fields (12 mice) were analysed per group. The goblet cells number per *μ*m^2^ were counted.

### 2.8. Colonic RNA and Fecal DNA Isolation and Quantitative RT-PCR Analysis

Specific operation is supplied in the Supplementary Material. The primers used are shown in Table 2.

### 2.9. Cell Culture and Treatment

We use 96-well culture plates (5 × 10^6^cells/mL) and 12-well culture plates (5 × 10^5^cells/mL) to culture the human colonic intestinal epithelial cells (HT-29, CL-0118, China). The LPS-treated cells (10 nM, Solarbio Ltd., Beijing, China) were treated with 100 *μ*M NAC (a ROS scavenger; MCE, New Jersey, USA; LPS + NAC-cells), 100 *μ*M TAK-242 (a TLR4 antagonist; MCE, New Jersey, USA; LPS + TAK-242-cells), 2 *μ*M TWS119 (a selective GSK-3*β* antagonist; MCE, New Jersey, USA; LPS + TWS-cells), or 10^−8^ M MT (Sigma-Aldrich, St. Louis, USA; LPS + MT-cells). After MT supplementation for 30 min, the LPS + MT-cells were sequentially treated with 50 *μ*M 4P-PDOT (a nonselective MT2 antagonist; MCE, New Jersey, USA; LPS + MT + 4P-PDOT-cells). Incubate each treated cells for 24 h. Meanwhile, some *Aeromonas* veronii-supplied cells (ATCC35624, *Aeromonas* cells) were treated with 10^−10^-10^−8^ M MT (*Aeromonas* + MT-cells). Each plate was inoculated with a bacterial suspension at a ratio of 1 : 5-5 : 1 (bacteria: host cells) for 0-12 h.

The IECs collected from the 96-well culture plates were detected for ROS (Nanjingjianchen, Beijing, China) assay and proliferation activity using MTT (3-(4,5)-dimethylthiahiazo (-z-y1)-3,5-di-phenytetrazoliumromide; Sigma, St. Louis, MO, USA) assay. A microplate reader (Model 680, Bio-Rad, St. Louis, MO, USA) equipped with a 570 nm wavelength filter was used to determine the optical density. Meanwhile, the cells collected from the 12-well culture plates was assessed for lactate dehydrogenase (LDH) assessment, quantitative RT-PCR, and western blotting analysis. Each operation used a repeat of 8 wells.

### 2.10. LDH Assessment

Consistent with the manufacturer's instructions, we use a LDH test kit (Solarbio Ltd., Beijing, China) to detected the cell supernatants. The test data were assessed at 450 nm wavelength via a microplate reader (Model 680, Bio-Rad, St. Louis, MO, USA) and were expressed as U/10^4^ cells. Every sample was assayed three times.

### 2.11. Determination of ROS Formation

Consistent with the manufacturer's instructions, we use a ROS test kit, purchased from Sigma-Aldrich to detect the ROS content (*n* = 9). A flow cytometer with an oxidation-sensitive DCFH-DA fluorescent probe was used to measure the intracellular ROS generation. Specific operation is supplied in the Supplementary Material.

### 2.12. Western Blotting

The appropriate amount of colon segment was quickly homogenized in liquid nitrogen and stored at -80°C refrigerator for western blotting analysis. Specific operation is supplied in the Supplementary Material.

### 2.13. Statistical Analysis of Data

We use SPSS 10.0 statistical software (SPSS, Inc., Chicago, IL, USA) to analysed the data and showed as themean ± standard error. Differences between groups were statistically analysed using one-way ANOVA, which was used to indicated the significance of differences among groups (*P* < 0.05 and *P* < 0.01).

## 3. Results

### 3.1. Melatonin Improved SD-Induced Aeromonas and LPS Increase and MUC2 Deficiency in Mice

**Figure 1:**
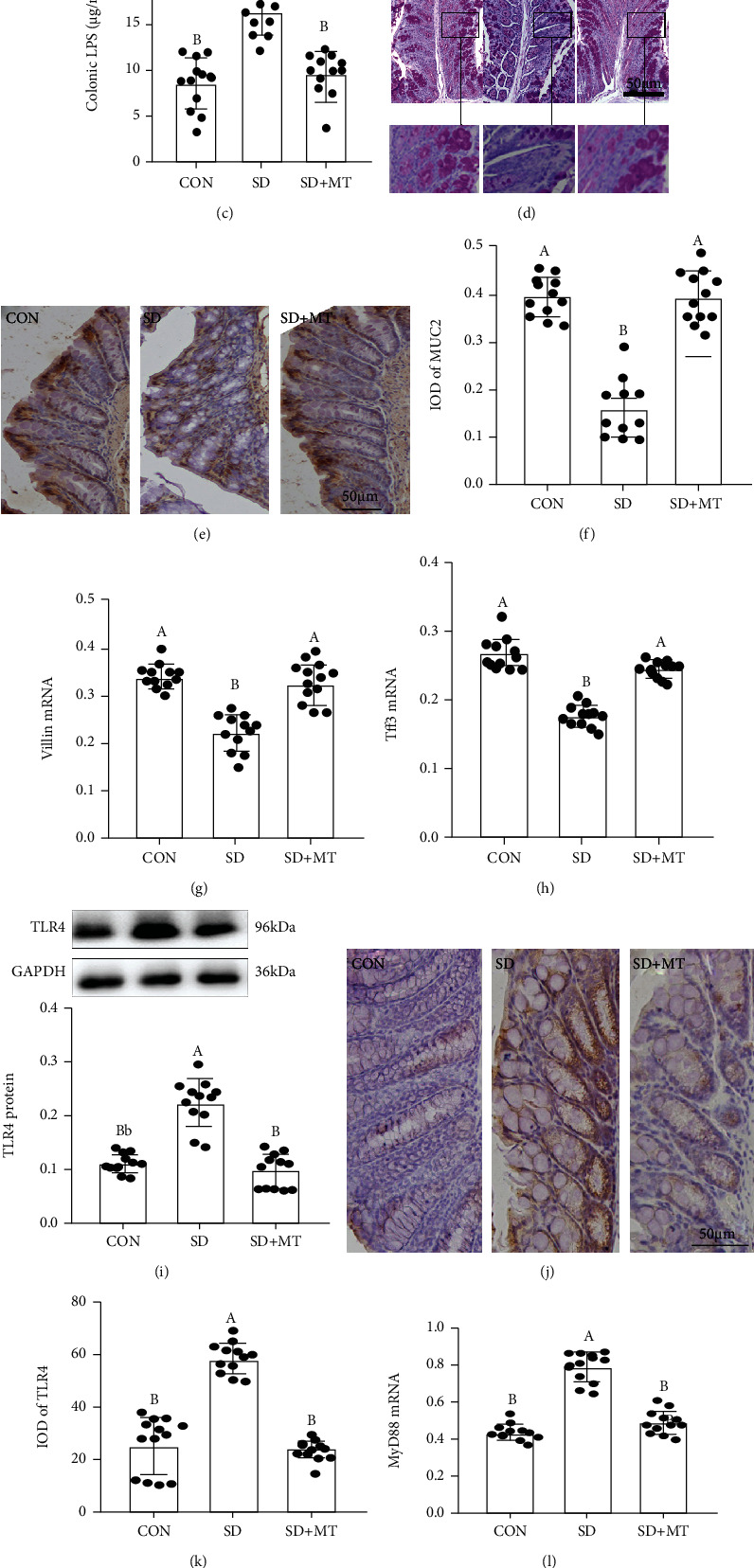
Melatonin improved SD-induced *Aeromonas* and LPS level increase and MUC2 deficiency in mice. (a) DAI score; relative abundance of colonic *Aeromonas* (b) and LPS (c); (d) PAS staining of colon tissue sections (scale: 50 *μ*m); (e) immunohistochemical staining of MUC2 in colon sections (scale: 50 *μ*m); (f) IOD of MUC2 protein; (g) Villin mRNA; (h) Tff3 mRNA; (i) TLR4 protein; (j) immunohistochemical staining of TLR4 in colon sections (scale: 50 *μ*m); (k) IOD of TLR4 protein; (l) MyD88 mRNA; (m) p-GSK-3*β* and (n) *β*-catenin proteins in CON, SD, and SD+MT groups. Values are presented as mean ± SE. Differences were assessed using ANOVA and are denoted as follows: different lowercase letters: *P* < 0.05; different uppercase letters: *P* < 0.01; same letter: *P* > 0.05. The bottom is the same.

Results showed the occurrence of colitis in SD mice, including weight loss (Figure S1A), colon shortening (Figures S1B, C), fecal occult blood (Figure S1F), the increase of histological score (Figures S1D, E), intestinal permeability (Figure S1G), and IL-17 levels (Figure S1H), compared with CON group. Our researches demonstrated that SD induced an elevation in the DAI score (Figure 1(a)). Moreover, there was an upregulation of relative abundance in colonic *Aeromonas* (Figure 1(b)) and LPS (Figure 1(c)) and a reduction in the goblet cells number (Figure 1(d)) in the SD group relative to the CON group. Meanwhile, compared with the CON group, there was a decrease in MUC2 protein (Figures 1(e) and 1(f)), Villin (Figure 1(g)), and Tff3 mRNA (Figure 1(h)). Furthermore, there was an upregulation in the expression levels of TLR4 (Figures 1(i–k)) and MyD88 (Figure 1(l)), and a reduction in the expression levels of p-GSK-3*β* (Figure 1(m)) and *β*-catenin (Figure 1(n)) in the SD groups relative to the CON group.

MT-supplied elevated the colitis phenotype, goblet cells number and expression level of MUC2, Villin, Tff3, p-GSK-3*β* and *β*-catenin and reduced the DAI score, level of Aeromonas and LPS, and contents of TLR4 and MyD88, and there was no obviously difference between the SD+MT and control groups (*P* > 0.063).

### 3.2. FMT Promotes Reestablishment of the Intestinal Microecology

**Figure 2:**
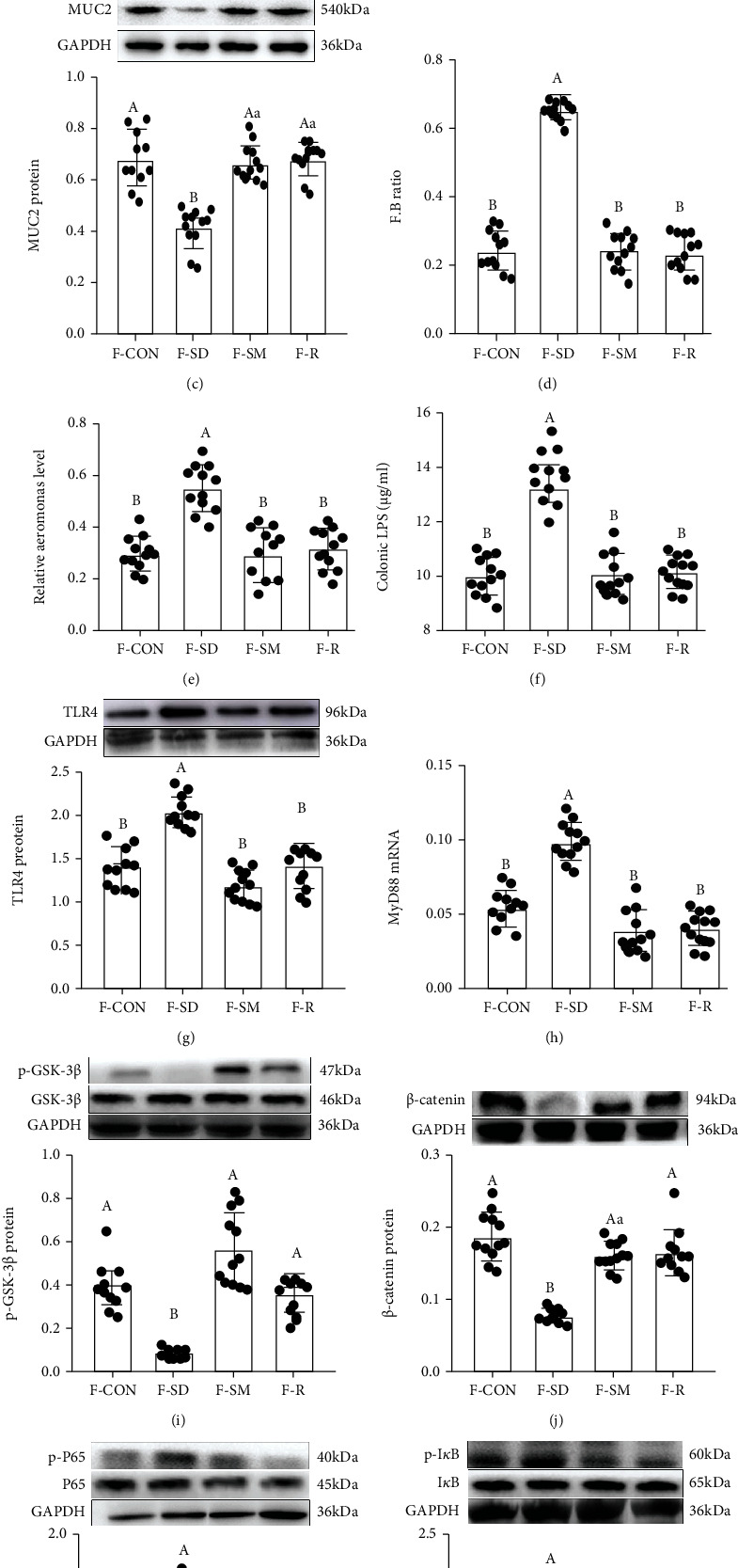
FMT reestablished the intestinal microecology similar to CON, SD, and SD+MT mice. (a) PAS staining of colon tissue sections (scale: 50 *μ*m); (b) the number of goblet cells per um^2^ in the colon; (c) MUC2 protein; (d) the ratio of F:B; Relative abundance of *Aeromonas* (e) and LPS (f) in the colonic content; colonic TLR4 (g), MyD88 (h), GSK-3*β* (i), *β*-cateinin (j), p-P65 (k), and p-I*κ*B (l) mRNA and proteins of the F-CON, F-SD, F-SM, and F-MT groups.

As illustrated in Figure 2, compared with the F-CON group, the body weight, colonic length, the level of IL-10, and IFN-*γ*, the goblet cells number and expression levels of MUC2, p-GSK-3*β*, and *β*-catenin proteins decreased by 2.3 ± 0.669% (Figure S2A), 56.8 ± 0.134% (Figures S2D, E), 42.3 ± 1.004% (Figure S2H), 34.2 ± 2.115% (Figure S2I), 4.3 ± 3.314% (Figures 2(a) and 2(b)), 54.3 ± 0.015% (Figure 2(c)), 48.9 ± 0.006% (Figure 2(i)), and 62.1 ± 0.002% (Figure 2(j)), respectively, while the intestinal permeability, histological score, the level of IL-1*β* and IL-6, F:B ratio, relative abundance of *Aeromonas* and LPS, and expression levels of TLR4, MyD88, p-P65, and p-I*κ*B proteins increased by 43.9 ± 0.006% (Figure S2C), 52.1 ± 0.007% (Figures S2F, G), 29.8 ± 0.150% (Figure S2J), 35.6 ± 0.009% (Figure S2K), 38.1 ± 0.343% (Figure 2(d)), 51.5 ± 0.009% (Figure 2(e)), 48.1 ± 0.221% (Figure 2(f)), 35.7 ± 0.005% (Figure 2(g)), 45.2 ± 0.008% (Figure 2(h)), 32.1 ± 0.004% (Figure 2(k)), and 26.9 ± 0.005% (Figure 2(l)) in the F-SD group, with fecal occult blood (Figure S2B). Moreover, there was an upregulation of the relative abundance in Firmicutes and proteobacteria (Figures S3B, C) and a downregulation of bacteroidetes and Faecalibacterium (Figures S3A, D) in F-SD group related to F-CON group. Yet, the stimulating effects of F-SD on changes in colitis and intestinal microbiota imbalance were improved in the colon via F-MT supplementation.

### 3.3. A. veronii Colonization Promoted the Occurrence of Colitis and MUC2 Deficiency in Mice

**Figure 3:**
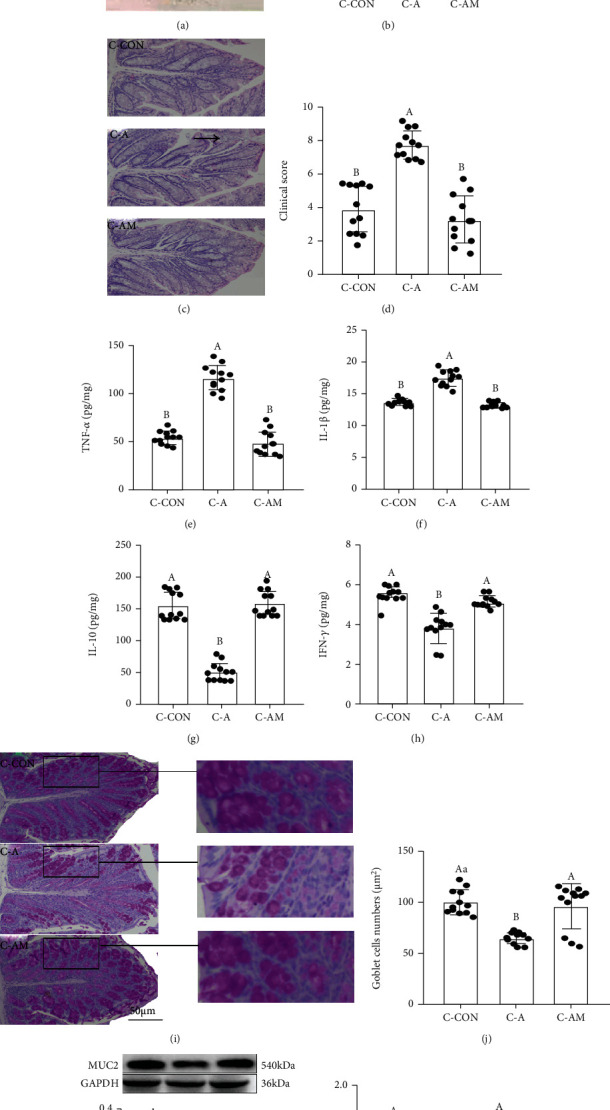
*Aeromonas* veronii colonization promoted the occurrence of colitis in mice. (a): fecal occult blood; (b) relative luciferase activity for colonic permeability; (c) H&E staining photographs (scale: 50 *μ*m); (d) histopathological score; colonic TNF-*α* (e), IL-1*β* (f), and IL-10 (g), and IFN-*γ* (h) concentrations were measured by ELISA; (i) PAS staining of colon tissue sections (scale: 50 *μ*m); (j) the number of goblet cells per um^2^ in the colon; (k) MUC2 protein; (l) Villin mRNA; (m) Tff3 mRNA in C-CON, C-A, and C-AM groups.

To verify the core role of the *Aeromonas* level increase in SD-induced MUC2 deficiency, we established an *A.* veronii colonization mouse model. After *A.* veronii colonization, we observed an upregulation of the relative level of *Aeromonas* and LPS by 31.2 ± 0.254%, *P* = 0.024 (Figure S4A) and 29.1 ± 0.061%, *P* = 0.025 (Figure S4B), respectively, and Pearson correlation analysis demonstrated a positive correlation between the relative content of *Aeromonas* and the colonic LPS level (*r*^2^ = 0.9131, *P* < 0.0001, Figure S4C) in mice. Moreover, the mice exhibited a more serious fecal occult blood level (Figure 3(a)), elevations of permeability (Figure 3(b)), and clinical score (Figures 3(c) and 3(d)) and levels of TNF-*α* (Figure 3(e)) and IL-1*β* (Figure 3(f)), as well as reductions of IL-10 (Figure 3(g)) and IFN-*γ* (Figure 3(h)) levels, goblet cells' number (Figures 3(i) and 3(j)), and expression levels of MUC2 (Figure 3(k)), Villin (Figure 3(l)), and Tff3 (Figure 3(m)), relative to the C-CON group. However, MT supplementation suppressed this process and caused no obvious differences between the C-CON and C-AM groups (*P* > 0.053).

### 3.4. Melatonin Ameliorates LPS-Induced Colitis

**Figure 4:**
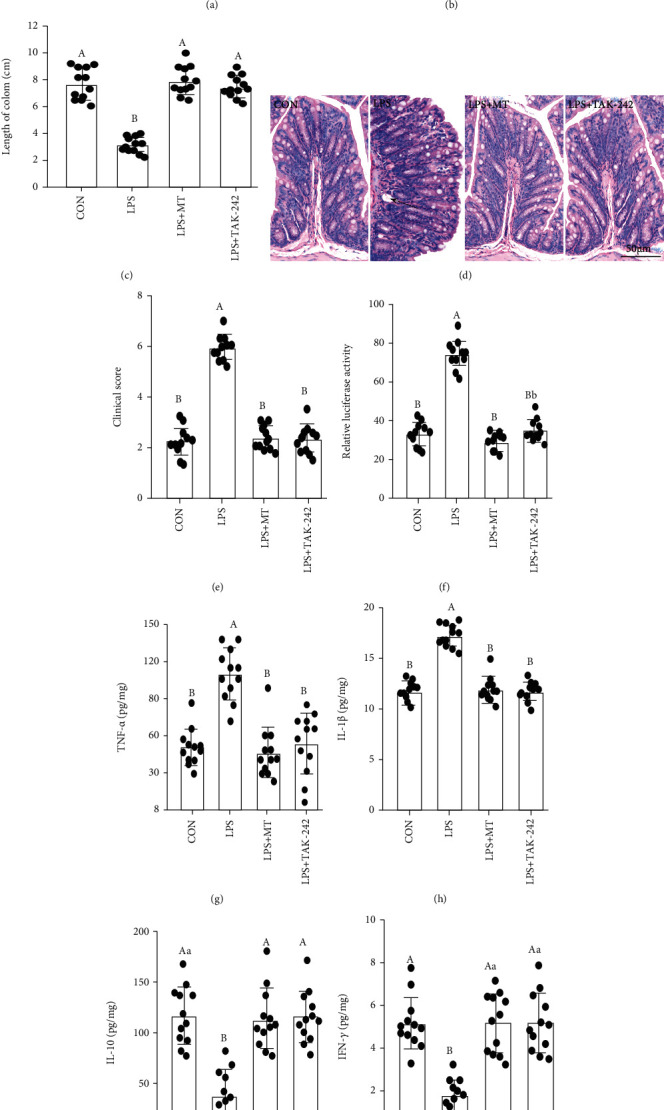
Melatonin improved LPS induced colitis in mice. (a) body weight; (b) and (c) colonic length; (d) H&E staining photographs (scale: 50 *μ*m); (e) histopathological score; (f) relative luciferase activity for colonic permeability; (g–j) TNF-*α* (g); IL-1*β* (h); IL-10 (i), and IFN-*γ* (j) concentrations were measured by ELISA in the colon of the CON, LPS, LPS+MT, and LPS+TAK-242 groups.

To evaluate the clinical relevance of MT and LPS-mediated mucin deficiency, we established a LPS-induced mouse colitis model with or without MT and TAK-242 supplementation. We observed no significant changes in liver morphology (Figure S5A) and levels of inflammatory factors (IL-6, TNF-*α*, IFN-*γ*, and IL-10, Figures S5B-E) in mice of CON, LPS and, LPS+MT groups. However, compared with the CON group, the LPS-treated group showed a decrease in body weight (Figure 4(a)), colon length (Figures 4(b) and 4(c)), IL-10 (Figure 4I), and IFN-*γ* (Figure 4(j)) as well as an upregulation in histopathological score (Figures 4(d) and 4(e)), permeability (Figure 4(f)), TNF-*α* (Figure 4(g)), and IL-1*β* (Figure 4(h)).

In contrast, MT and TAK-242 supplementation reversed the LPS-induced changes in colitis and the inflammatory response; no significant difference was showed in body weight (*P* > 0.052), colonic length (*P* > 0.556), colonic permeability (*P* > 0.345), histopathological score (*P* > 0.643), proinflammatory cytokines (TNF-*α* and IL-1*β*, *P* > 0.772), or anti-inflammatory factors (IL-10 and IFN-*γ*, *P* > 0.558) among the LPS+MT, LPS+NAC, LPS+TAK-242, and CON groups.

### 3.5. Melatonin Ameliorates LPS-Induced MUC2 Depletion and Changes in the Expression Levels of Signalling Proteins in Mice

**Figure 5:**
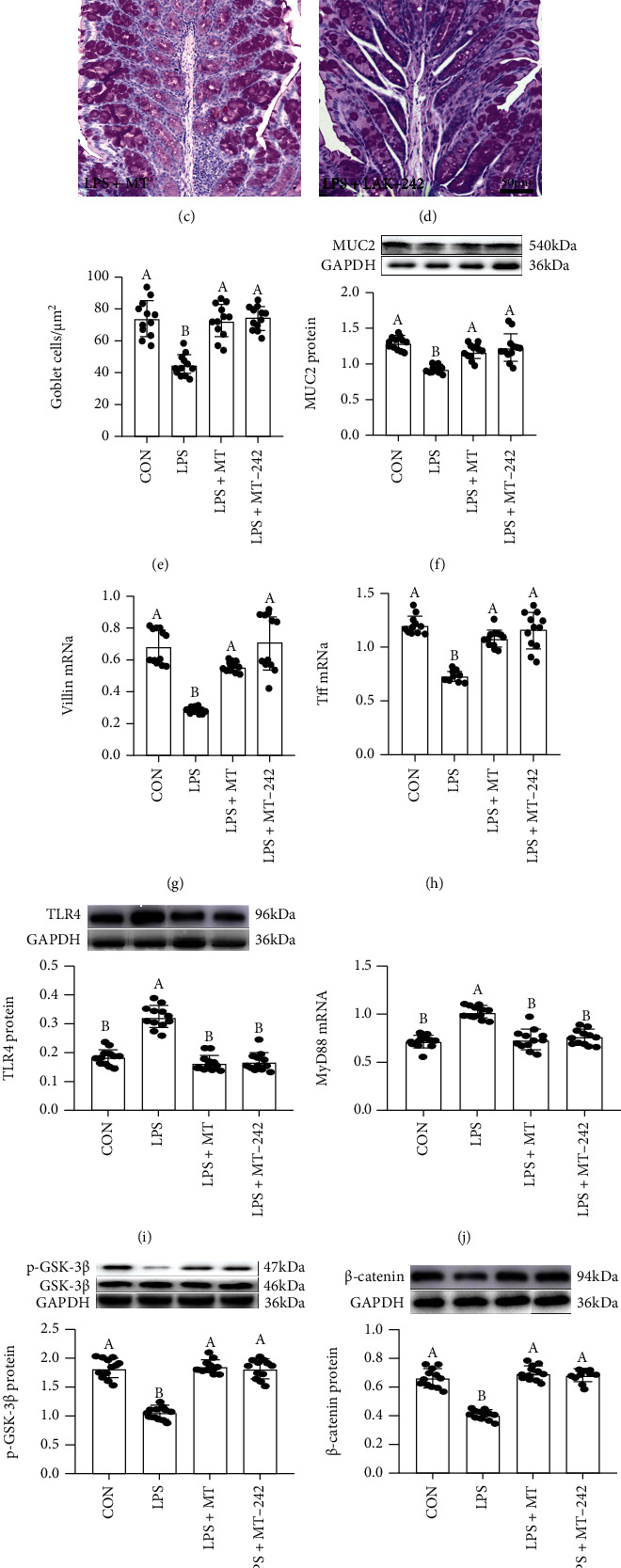
Melatonin improved LPS-induced MUC2 deficiency and the changes of expression levels in signalling proteins in mice. (a–d) PAS staining of colon tissue sections (scale: 50 *μ*m); (e) the number of goblet cells per um^2^ in the colon; (f) colonic MUC2 protein; (g) Villin, and (h) Tff3 mRNA; (a) colonic TLR4, (b) MyD88, (c) p-GSK-3*β*, (d) *β*-catenin, (e) p-I*κ*B, and (f) p-P65 proteins and mRNA production in the CON, LPS, LPS+MT, and LPS+TAK-242 groups.

Meanwhile, the PAS staining consequence indicated that the goblet cells number was significantly reduced by 40.6 ± 2.513% (*P* = 0.035) in the colon in the LPS group (Figures 5(a)–5(e)). An analogous effect was presented in the expression levels of MUC2 protein, Villin, and Tff3 mRNA, which was significantly decreased by 20.6 ± 0.019% (MUC2, *P* = 0.009, Figure 5(f)), 48.3 ± 0.007% (Villin, *P* ≤ 0.001, Figure 5(g)), and 36.4 ± 0.018% (Tff3, *P* = 0.007, Figure 5(h)), respectively, in the LPS group related to the control group. While these diversifications were improved after MT and TAK-242 supplementation, leading to no obviously difference among these groups (*P* > 0.209).

Moreover, our researches indicated that there was an increase in the expression levels of TLR4 (Figure 5(i)), MyD88 (Figure 5(j)), p-I*κ*B (Figure 5(m)), and p-P65 (Figure 5(n)) and a decrease in p-GSK-3*β* (Figure 5(k)) and *β*-catenin (Figure 5(l)) in the LPS group compared with the CON group. While after MT and TAK-242 supplementation, the expression levels of TLR4, MyD88, p-I*κ*B, and p-P65 proteins downregulated by 28.7 ± 0.020 − 35.7 ± 0.025%, 36.1 ± 0.039 − 47.3 ± 0.028%, 29.4 ± 0.047 − 48.9 ± 0.044%, and 25.4 ± 0.027 − 37.5 ± 0.030%, respectively, while the expression levels of p-GSK-3*β* and *β*-catenin proteins increased by 25.4 ± 0.039 − 32.9 ± 0.054% and 21.8 ± 0.050 − 33.6 ± 0.030%, respectively, relative to those in the LPS group, leading to no significant diversity between the CON group and the MT and TAK-242-supplied groups. Moreover, the changing trends of these proteins (TLR4, MyD88, p-GSK-3*β*, *β*-catenin, p-P65, and p-I*κ*B) are consistent with the mice in C-CON, C-A, and C-AM groups (Figure S6).

### 3.6. Melatonin Regulates the MUC2 Level in HT-29 Cells Treated with A. veronii

To determine the vital effect of MT in MUC2 production, we selected mucin-secreting human HT-29 cells exposed to various proportions (1 : 5~5 : 1) of *A.* veronii for 12 h, which formed a homogeneous population of polarized goblet cells. Compared to the cells in the CON group, *A.* veronii treatments from 1 : 3-5 : 1 significantly inhibited the proliferation activity and MUC2 expression level of HT-29 cells, which decreased by 44.2 ± 0.010 − 56.9 ± 0.007% (Figure S7A) and 38.6 ± 0.011 − 62.1 ± 0.005% (Figure S7D), respectively. A decrease in proliferation activity and MUC2 level was examined after 8 h of infiltrated with 1 : 1 *A.* veronii, which reduced by 35.2 ± 0.009% (Figure S7B) and 34.1 ± 0.040% (Figure S7E), respectively. To investigate the molecular pathway via which MT regulates colonic MUC2 production, cells were supplied with MT at 10^−10^-10^−8^ M concentrations 1 h prior to *A.* veronii treatment (1 : 1) for 8 h. We observed that the proliferation activity and expression level of MUC2 protein were decreased by 54.8 ± 0.007% (Figure S7C) and 61.2 ± 0.033% (Figure S7F) in the *A.* veronii-treated group relative to the CON group, respectively. Obviously, the inhibitory effect of *A.* veronii on proliferation activity and MUC2 expression was reversed by pretreatment with MT after 10^−8^ M MT supplementation.

### 3.7. Regulatory Effect of Melatonin on the TLR4/MyD88/GSK-3*β*/*β*-catenin/NF-*κ*B Loop in A. veronii-Treated HT-29 Cells

**Figure 6:**
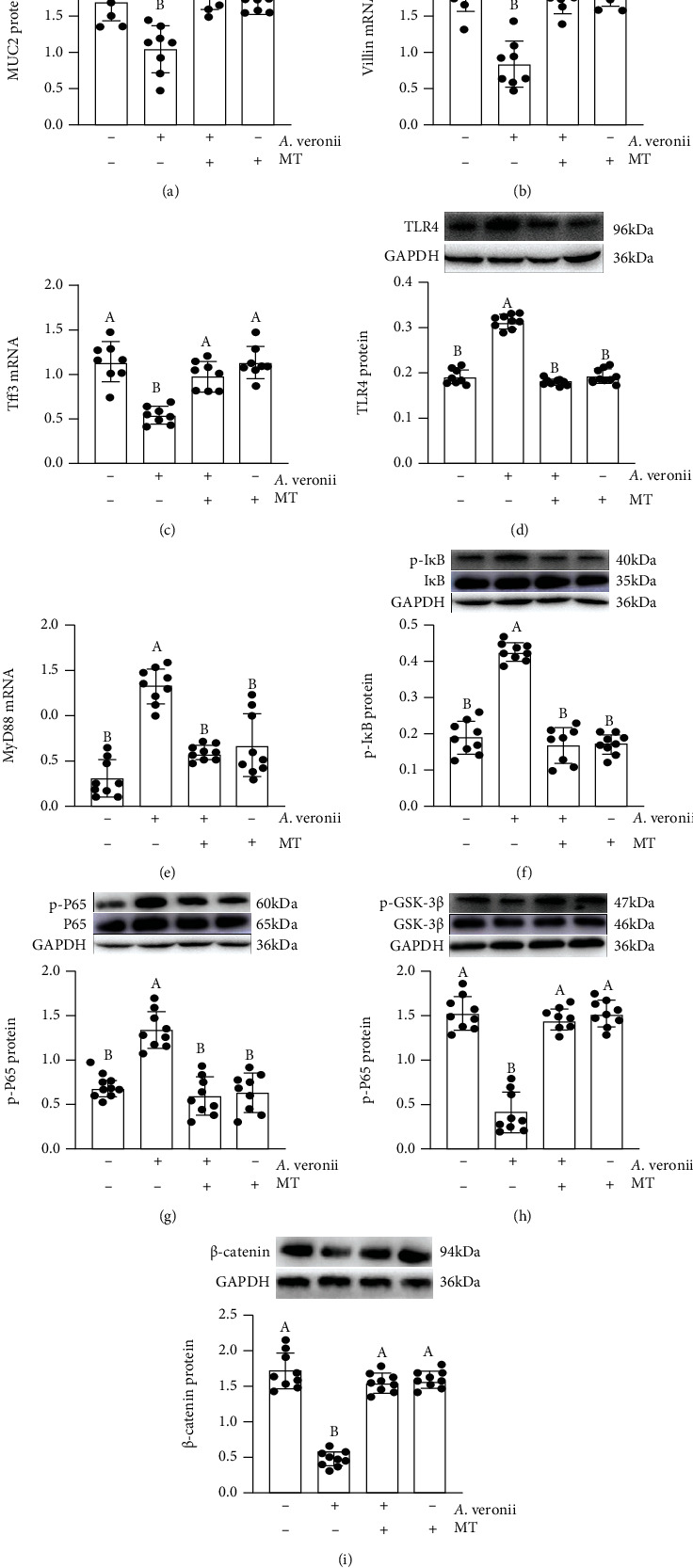
Melatonin suppressed the changes in expression levels of signalling proteins in *Aeromonas*-treated HT-29 cells. MUC2 (a), Villin (b), Tff3 (c), TLR4 (d), MyD88 (e), p-I*κ*B (f), p-P65 (g), p-GSK-3*β* (h) and *β*-catenin (i) proteins, and mRNA in various treatment groups.

The results demonstrated that there was an obviously reduction in the expression levels of MUC2 (Figure 6(a)), Villin (Figure 6(b)), Tff3 (Figure 6(c)), p-GSK-3*β* (Figure 6(h)), and *β*-catenin (Figure 6(i)) and an upregulation in the expression levels of TLR4 (Figure 6(d)), MyD88 (Figure 6(e)), p-I*κ*B (Figure 6(f)), and p-P65 (Figure 6(g)) in the *A.* veronii-treated group related to the vehicle group. While MT supplementation effectively improved these *A.* veronii-induced changes. In contrast, we observed a downregulation of TLR4, MyD88, p-I*κ*B, and p-P65 and an upregulation of MUC2, Villin, Tff3, p-GSK-3*β*, and *β*-catenin related to the *A.* veronii-treated group.

### 3.8. Melatonin Regulates the MUC2 Level in HT-29 Cells Treated with LPS

To investigate the effect of LPS in the regulation of mucin expression levels, HT-29 cells were infiltrated with different concentrations (0-500 pg/mL) of LPS for 24 h. Compared to the cells in the CON group, LPS-treated from 100 to 500 pg/mL obviously suppressed the mucin expression levels of HT-29 cells (Figure S8A), which decreased by 54.3 ± 0.084% and 58.4 ± 0.084%, respectively. A reduction in MUC2 expression level was presented after 24 h of exposed to 100 pg/mL LPS (Figure S8B), which was reduced by 35.2 ± 0.078%. To verify the molecular pathways by which MT regulates colonic MUC2 production, the cells were treated with 10^−8^ M MT thirty min prior to LPS (100 pg/mL) for 24 hours. We observed that the expression level of MUC2 protein was decreased by 12.3 ± 0.065% in the LPS-treated group relative to the CON group. Obviously, the inhibitory effect of LPS on MUC2 expression was reversed by supplementation with MT (Figure S8C). We also explored the ability of MT to improve cell viability in LPS-treated IECs in an LDH assay. Compared with the control cells, LPS caused a large amount of cell death and released LDH (Figure S8D). Statistically, the LDH level was obviously upregulated (Figure S8E) in the LPS-treated group relative to the vehicle group. Conversely, the MTT assay indicated that LPS caused a decrease in proliferation index (Figures S8F, G). However, after MT supplementation, the LDH index was significantly reduced by 12.5 ± 0.094%, while the proliferation capacity observably increased by 17.1 ± 0.075%, which almost returned to the vehicle level.

### 3.9. Melatonin Inhibited TLR4/MyD88 Pathway-Mediated Oxidative Stress Activation-Induced MUC2 Depletion in HT-29 Cells Treated with LPS

**Figure 7:**
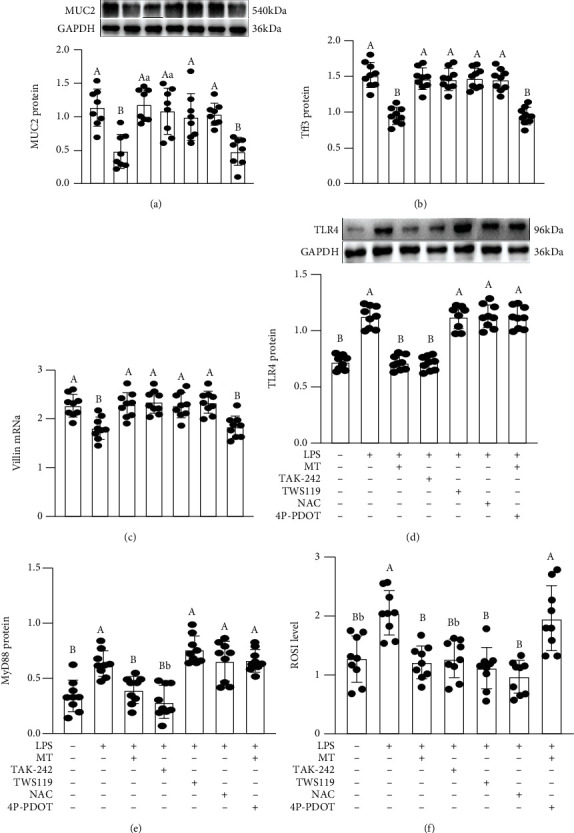
Melatonin suppressed the changes in expression levels of signalling proteins (MUC2, Tff3, Villin, TLR4, and MyD88) in LPS-treated HT-29 cells. MUC2 (a), Tff3 (b), Villin (v), TLR4 (d), MyD88 (e) proteins and mRNA content and ROS (f) in various treatment groups. NAC: ROS scavenger; TAK-242: an antagonist of TLR4; TWS119: an antagonist of GSK-3*β*; 4P-PDOT: an antagonist of MT2. The bottom is the same.

Consistent with the anticipation, LPS-treated obviously caused a decrease in the expression levels of MUC2 protein (Figure 7(a)), Tff3 (Figure 7(b)), and Villin mRNA (Figure 7(c)) as well as an upregulation in the expression levels of TLR4 protein (Figure 7(d)), MyD88 mRNA (Figure 7(e)), and ROS content (Figure 7(f)) compared with the vehicle group. By contrast, MT-supplied improved the stimulatory effect of LPS, leading to no significant difference between the CON group and the MT-supplied group. Moreover, the LPS-induced effect was blocked by pretreatment with TAK242, which showed an upregulation of MUC2, Tff3, and Villin mRNA and a downregulation of TLR4, MyD88, and ROS contents in the LPS+TAK242-treated IECs relative to the LPS-treated group. Similarly, TW119 supplementation effectively reversed these LPS-induced changes, while it had no effect on the expression levels of TLR4 and MyD88. Furthermore, our results demonstrated that NAC imitated the improvement effect of MT and upregulated the expressions of MUC2 protein, Tff3, and Villin mRNA, as well as downregulated the ROS content in LPS+NAC-treated IECs relative to the LPS-treated group. Yet, it had no influence on the expression levels of TLR4 and MyD88. These studies indicated that the inhibitory influence of MT on oxidative stress closely associates with the suppression of TLR4/MyD88 pathway induced by LPS. In contrast, 4P-PDOT pretreatment counteracted the effects of MT. The expressions of MUC2, Tff3, and Villin were decreased by 33.9 ± 0.081%, 35.9 ± 0.040%, and 32.9 ± 0.076%, while TLR4, MyD88, and ROS contents were increased by 42.9 ± 0.064%, 35.2 ± 0.070%, and 38.9 ± 0.092% in the LPS+MT+4P-PDOT group versus the LPS+MT group.

### 3.10. Regulatory Effect of Melatonin on the GSK-3*β*/*β*-catenin/NF-*κ*B Loop Mediated by TLR4/MyD88 Pathway Inactivation in HT-29 Cells Treated with LPS

**Figure 8:**
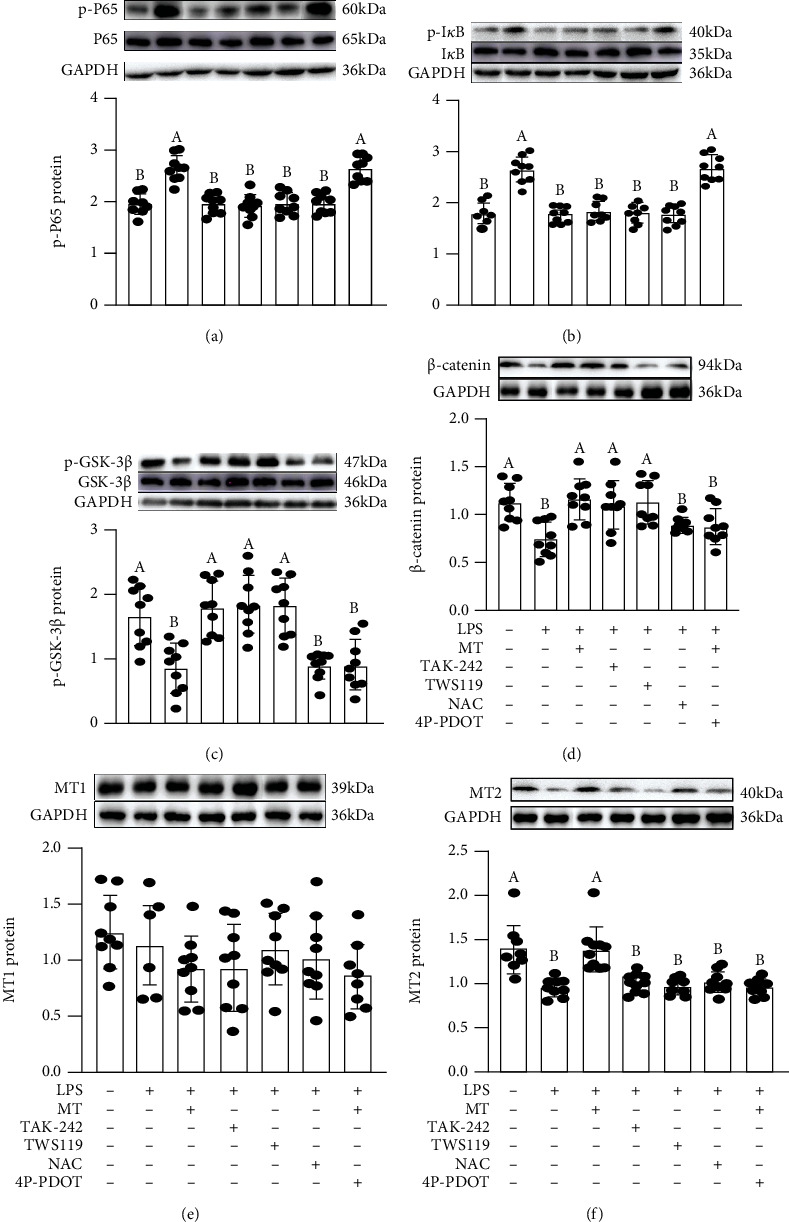
Melatonin suppressed the changes in expression levels of signalling proteins (p-P65, p-I*κ*B, p-GSK-3*β*, *β*-catenin, MT1, and MT2) in LPS-treated HT-29 cells. p-P65 (a), p-I*κ*B (b), p-GSK-3*β* (c), *β*-catenin (d), MT1 (e), and MT2 (f) proteins in various treatment groups.

Next, we explored how TLR4/MyD88 activation was related to mucin deficiency. The results showed an upregulation in the expression levels of p-P65 (Figure 8(a)) and p-I*κ*B (Figure 8(b)) and a significant reduction in the expression levels of p-GSK-3*β* (Figure 8(c)), *β*-catenin (Figure 8(d)), and MT2 (Figure 8(f)) in the LPS-treated group, relative to that in the vehicle group while the expression level of MT1 protein did not change (Figure 8(e)). While MT supplementation effectively improved these LPS-induced changes. In contrast, after treatment with TAK-242 and TWS119, we observed a downregulation of p-P65 and p-I*κ*B and an upregulation of p-GSK-3*β* and *β*-catenin relative to the LPS group while these had no influence on the expression of MT2 protein level. Moreover, our results suggested that NAC, imitated the improvement effect of MT and downregulated the expressions of p-P65 and p-I*κ*B proteins in LPS+NAC-treated IECs relative to the LPS-treated group while it had no influence on the expression levels of p-GSK-3*β*, *β*-catenin, and MT2 proteins. However, in treatment with 4P-PDOT, the antagonist of MT2 counteracted the therapeutic influences of MT and failed to reverse the changes induced by LPS, which further confirmed the role of the TLR4/MyD88-mediated GSK-3*β*/*β*-catenin/NF-*κ*B/ROS pathway in LPS-treated IECs.

## 4. Discussion

Our previous results showed that SD induced intestinal mucosal barrier damage, including a decrease in the number of goblet cells and MUC2 protein and an upregulation of pathogen (*Aeromonas*) content [18]. Meanwhile, FMT from SD mice to wild mice recapitulates the SD-like *Aeromonas* content increase and goblet cells number and MUC2 protein level decreases, while MT improved these changes. These results demonstrated a high correlation between *Aeromonas* level increase and goblet cells number decrease. In the SD experiment, we found that the relative abundance of *Aeromonas* in the colon of the SD mice was upregulated most significantly, and after melatonin supplementation, it returned to the control level. *Aeromonas* species belong to Gram-negative microbiota usually resides in the intestines. Members of the genus *Aeromonas* are related to lots of interintestinal and extraintestinal infections in rodents [22]. *Aeromonas* obviously induces intestinal inflammation, while can frequently cause extraintestinal inflammatory responses, e.g., biliary system infection, necrotizing fasciitis, cholangitis, surgical wounds, abdominal meningitis, and posttraumatic cellulitis [23]. To test the vital role of *Aeromonas* in SD-induced MUC2 depletion, we established an *A.* veronii colonization mouse model. The results indicated that *A.* veronii-colonized mice exhibited a colitis phenotype, including an upregulation of *Aeromonas* and its cell wall composition LPS levels and a downregulation of goblet cells' number and MUC2 protein level. However, MT supplementation significantly suppressed *A.* veronii colonization-induced proliferation of *Aeromonas* and LPS, restored MUC2 deficiency, and improved colitis, which strongly indicated that *Aeromonas* coupling with goblet cells promoted MUC2 depletion, further inducing colitis.

Furthermore, we set up a LPS-treated mouse model with or without MT supplementation to demonstrate that excessive *Aeromonas*-related LPS has a negative effect on MUC2 depletion in SD mice. The study is consistent with previous researches in mice that were exposed to LPS that caused obvious signs of colitis: shorter colonic length, lighter weight, and more serious intestinal permeability [24]. Meanwhile, there was an elevation in LPS level and a reduction in goblet cells number and MUC2 protein, Tff3, and Villin mRNA expression levels in LPS-treated mice. Importantly, the decrease of goblet cells number, MUC2 content, and a thinner intestinal mucus layer closely associates with IBD [9]. Van der Sluis et al. previously pointed that MUC2^−/−^ mice behave with clinical and histological characteristics of colitis [25]. Furthermore, the present findings also showed that MT supplementation could alleviate LPS-induced colitis and the reduction of goblet cells and MUC2 protein, Villin, and Tff3 mRNA. Similarly, Shah et al. showed that MT could promote the proliferation of goblet cells to secrete mucin to resist pathogenic bacteria [26]. Therefore, we speculated that MT could improve colitis by suppressing *Aeromonas-*goblet cell interactions, thereby upregulating the goblet cells number and MUC2 protein, further improving SD-induced colitis. Meanwhile, we observed a decrease in the expression levels of p-GSK-3*β* and *β*-catenin proteins, as well as an increase in TLR4, MyD88 and p-P65, and p-I*κ*B proteins in LPS-treated mice, while MT supplementation restored these changes. Similarly, TAK-242 (an antagonist of TLR4) supplementation mimicked the improvement effect of MT. Researches demonstrated that inappropriate TLR activation can induce prolonged inflammatory response and even autoimmune and inflammatory diseases [27]. Similarly, in rat biliary epithelia, anti-TLR2 antibodies or anti-TLR4 antibodies supplementation could reduce MUC2 expression treated with LPS [28]. It seems that boosting LPS activates the TLR4-MyD88-GSK-3*β*-NF-*κ*B pathway and promotes MUC2 depletion, further inducing colitis.

In vitro, we further found that LPS and *A.* veronii could upregulate the LDH index and ROS abundance and downregulate the expression of MUC2 protein level and cell proliferation activity in mucus-secreting human HT-29 cells. However, MT supplementation effectively suppressed this process. Further, the ameliorate effect of MT could be suppressed by 4P-PDOT. However, TAK242 mimicked the effect of MT in LPS-treated cells, while it had no influence on the expression level of MT2 proteins. LPS binds innate immunity TLRs, which activates signalling cascades related to NF-*κ*B transcription factor and mitogen-activated protein kinases (MAPKs) in adipocytes [29]. Furthermore, we found that TWS119 treatment enhanced the expressions levels of p-GSK-3*β* and *β*-catenin, ultimately suppressing NF-*κ*B activation and promoting MUC2 secretion, while it had no influence on the expression levels of TLR4 and MT2 protein and MyD88 mRNA. Emerging date has also indicated that GSK-3*β* mediated the activation of the NF-*κ*B signalling cascade via enhancing the NF-*κ*B transcriptional activity in the nucleus to promote cancer [30]. Similarly, NAC counteracted the effect of LPS in HT-29 cells, which suppressed the activation of NF-*κ*B pathway. Previous researches indicated that oxidative stress-mediated NF-*κ*B activation ultimately relies on the phosphorylation and proteasomal degradation of its inhibitor I*κ*B*α*, supporting nuclear NF-*κ*B translocation. Therefore, in our study, a large number of in vivo and in vitro experiments have been demonstrated that melatonin-mediated MT2 inhibits aeromonas-goblet cell interactions to restore the level of MUC2 production via the LPS/TLR4/MyD88/GSK-3*β*/ROS/NF-*κ*B loop, further improving colitis in SD mice. Certainly, additional loss-of-function experiments using knock-down or knock-out mice/cell lines would be useful.

## 5. Conclusions

Overall, our study revealed that MT2-mediated MT suppressed *Aeromonas* coupling with goblet cells and restored MUC2 depletion by inhibiting the TLR4/MyD88/GSK-3*β*/*β*-catenin/ROS/NF-*κ*B loop, ultimately improving SD-induced colitis in mice. Obviously, the study supported original evidence for the useful influence of MT as a physiological controller of *Aeromonas*-induced MUC2 deficiency and supported the recent enlarging of the definition of psychobiotics to include MT-based strategies.

## Figures and Tables

**Table 1:** DAI score evaluation.

Weight loss	Score	Blood in stool	Score	Stool consistency	Score
**<**1%	0	Absence	0	Normal	0
1-5%	1		1	Soft stools	1
5-10%	2	Slight bleeding	2	Loose stools	2
10-15%	3		3	Mild diarrhea	3
**>**15%	4	Gross bleeding	4	Watery diarrhea	4

**Table 2:** Primers of target genes and reference gene.

Gene	Sense	Antisense
*MyD88*	CCTGCGGTTCATCACTAT	GGCTCCGCATCAGTCT
*MUC2*	CTGCACCAAGACCGTCCTCATG	GCAAGGACTGAACAAAGACTCAGAC
*Tff3*	GGCTGCTGCTTTGACTC	AGCCTGGACAGCTTCAA
*Villin*	TCGGCCTCCAGTATGTAG	CGTCTTCGGGGTAGAACT
*GAPDH*	CCGAGAATGGGAAGCTTGTC	TTCTCGTGGTTCACACCCATC
*Firmicutes*	GGAGCATGTGGTTTAATTCGAAGCA	AGCTGACGACAACCATGCAC
*Bacteroidetes*	GAGAGGAAGGTCCCCCAC	CGCTACTTGGCTGGTTCAG
*Proteobacteria*	GGTTCTGAGAGGAGGTCCC	GCTGGCTCCCGTAGGAGT
*Aeromonas*	AGAGTTTGATCCTGGCTCAG	GGCTACCTTGTTACGACTT
*Escherichia coli*	GGAGCAAACAGGATTAGATACCC	AACCCAACATTTCACAACACG

## Data Availability

All data generated or analysed during this study are included in this published article.

## Supplementary Materials

Supplementary Materials See supplementary methods and Figures S1-S8 in the Supplementary Material for comprehensive image analysis.
