# Supplementary material for: The Role of Aeromonas-Goblet Cell Interactions in Melatonin-Mediated Improvements in Sleep Deprivation-Induced Colitis
Source: Oxid Med Cell Longev. 2022 Mar 20;2022:8133310. doi: 10.1155/2022/8133310 (PMC8958064; doi:10.1155/2022/8133310)

**Materials and Methods**

**Animal Model Establishment**

Experiment 1

*Sleep Deprivation*. Continuous SD of the mice began at 8 am every day for 3 days using a modified multiple platform water bath as described previously [18]. Continuous sleep deprivation of the mice began from 8 am for 3 days using a modified multiple platform water bath. Eighteen platforms were placed in a water tank. Twelve mice were placed in the water bath. Every mouse in the water bath could move from one platform to another by jumping. The water filled the water bath 4 cm from the base. When the mice had reached the rapid eye movement stage of sleep, which is the paradoxical phase of sleep, muscle atonia could cause the mice to fall into the water. The mice then woke and would try to climb up the platform to avoid being drowned. Throughout the experiments, the water was replaced with clean water in the tank. Briefly, MT (M5250; Sigma, St. Louis, MO, USA) was dissolved in approximately 20 µL of absolute ethanol and diluted with 0.1 mL saline. CON mice were intraperitoneally injected with vehicle (0.1 mL saline containing 20 μL of absolute ethanol). SD-treated mice were administered intraperitoneal injections of MT 0 mg/kg (SD) and 20 mg/kg (SD+MT) once, 60 min before SD, and a single dose per day at 7:00 am for a total of 3 days. Mice are nocturnal animals, which are active at night and sleep during the day. Therefore, we imitated natural light to give the mice light at 7 o'clock to promote the mice to wake up and start sleep deprivation at 8 o'clock. Moreover, the melatonin was injected intraperitoneally at 7:00 am every day during 3 days of sleep deprivation. The time of melatonin supplementation was selected based on the published report, which rats were given to intraperitoneal injection of melatonin at a dose of 15 mg/kg/day (body weight) between 7:00 and 8:00 to explore the role of melatonin in ameliorating cognitive impairment induced by sleep deprivation.^19^ In addition, circadian expression of melatonin is affected by light. Peak melatonin secretion occurs during the daily dark period, while the melatonin concentration is low during the day. So we chose to give the sleep-deprived mice a timely melatonin supplementation at 7:00 am to exert beneficial effects of melatonin.

Colitis was assessed daily on the basis of the combined scores of body weight, stool consistency and presence of macroscopic blood in stools to calculate the disease activity index (DAI), as shown in Supplementary Table S1. The DAI score was
graded on a scale of 0–4, as previously described by Murthy et al [21].

Table S1. DAI score evaluation

| Weight loss | Score | Blood in stool | Score | Stool consistency | Socre |
| --- | --- | --- | --- | --- | --- |
| ＜ 1% | 0 | Absence | 0 | Normal | 0 |
| 1-5% | 1 |  | 1 | Soft stools | 1 |
| 5-10% | 2 | Slight bleeding | 2 | Loose stools | 2 |
| 10-15% | 3 |  | 3 | Mild diarrhea | 3 |
| ＞ 15% | 4 | Gross bleeding | 4 | Watery diarrhea | 4 |

Experiment 2

*Fecal Material Preparation and FMT Regime.* Fecal material was collected from mice in the CON, SD and SD+MT groups in the SD experiment, placed into Eppendorf tubes containing freezing solution (sterile saline solution with 12.5% glycerol). FMT was performed by oral gavage of a fecal slurry into naïve mice as described Stebegg et al [20]. Recipient lean mice were fasted for 2 h prior to FMT, and the fecal slurry were obtained from fecal pellets of 12 donor mice suspended by vortexing in 1 mL PBS per 100 mg of feces. Fecal mixtures were centrifugated at 500 g for 5 min and the supernatants were collected for FMT. The suspended pellets were then stored at -80 °C until utilized. For FMT, mice were randomized into the following groups: F-CON (antibiotic treatment followed by FMT from the CON group), F-SD (antibiotic treatment followed by FMT from the SD group), F-SM (antibiotic treatment followed by FMT from the SD + MT group) and F-R (vehicle). Before FMT, for substantial depletion of the microbiota, the mice (F-CON, F-SD and F-SM) were provided with drinking water containing 1 g/kg ampicillin (Santa Cruz Biotechnology, Delaware Ave., USA), 100 mg/kg gentamicin (Sigma-Aldrich, St. Louis, MO, USA), 0.5 g/kg neomycin (Sigma-Aldrich, St. Louis, MO, USA), 0.5 g/kg vancomycin (Hexal, Germany), and 10 mg/kg erythromycin (Sigma-Aldrich, St. Louis, MO, USA) for 10 days. FMT was carried out via oral gavage with a fecal suspension (100 mg/mL) (F-CON, F-SD and F-SM) or vehicle (F-R) in a final volume of 0.1 mL. FMT was performed continuously for 14 days per day AM 7:30 after 10 continuous days of antibiotic treatment.

Experiment 3

All mice were provided drinking water containing 2 mg/mL streptomycin (Santa Cruz Biotechnology, Delaware Ave., USA) to eliminate the normal microbiota for 5 days. Mice (C-A and C-AM) were then orally gavaged with 10^8^ CFU of organisms (in 0.1 mL PBS) at 8 am on the sixth day. The CON group was orally gavaged with vehicle (0.1 mL PBS). For treatment with MT (C-AM), 20 mg/kg MT was administered orally to mice by gavage once, 60 min before *Aeromonas* veronii colonization.

Experiment 4

LPS (27840; Sigma, St. Louis, MO) was dissolved in dimethyl sulfoxide (DMSO) and diluted in saline to a final concentration before injection. The LPS-supplemented mice were administered intraperitoneal injections of LPS 2 mg/kg every 3 days, a single dose per day at 8:00 am. For treatment with MT and TAK-242, 20 mg/kg/every 3 days MT (LPS+MT) and 10 mg/kg/every 3 days TAK-242 (TLR4 inhibitor, i.p.; Med Chem Express, LPS+TAK-242) were administered orally to mice by gavage once, 60 min before LPS supplementation, and a single dose per day at 7:00 am for a total of 3 days. After the injection, the mice were placed in cages and allowed to eat and drink freely.

All mice were euthanized under anesthesia using 10% chloral hydrate after the experiment ended at 8:00 am. Their plasma, colonictissue and colonic content were harvested.

*Faecal Occult Blood Test*. Apply a small amount of feces to the center of the glass slide and add 3 drops of 10g/L methylaminophenol sulfate solution and 3 drops of 3% hydrogen peroxide solution. Observe the results and take pictures immediately. *Histological Staining*. The colon was removed and placed in 4% paraformaldehyde containing 0.1 M phosphate buffered saline (pH 7.4, 4°C) for forty-eight hours, then infiltrated in paraffin. Hematoxylin and eosin (H&E) is used for histological staining. A microscope (BX51; Olympus, Tokyo, Japan) was used to randomly shoot at least 30 fields of view in 6 sections of each tissue, and analyze at least 180 fields of view for each sample.

*Immunohistochemical Staining.* We used immunohistochemistry to stain for MUC2 and TLR4 in paraffin intestinal sections. Sections were incubated overnight at 4°C with the monoclonal rabbit anti-mouse primary antibody (MUC2, 1:500; TLR4, 1:200; Abcam, Cambridge, MA, USA). Then, the sections were rinsed with 0.01 M PBS (pH 7.4) and incubated with biotinylated goat anti-rabbit IgG (1:200; Sigma, St. Louis, MO) for 2 h at room temperature. After washing, the tissues were incubated with streptavidin-horseradish peroxidase (1:250, Sigma, St. Louis, MO) for 2 h at room temperature. Immunoreactivity was visualized by incubating the tissue sections in 0.01 M PBS containing 0.05% 3’, 3-diaminobenzidine tetrahydrochloride (DAB; Sigma, St. Louis, MO) and 0.003% hydrogen peroxide for 10 min in the dark. The sections were then stained with haematoxylin and mounted. Control slides without the primary antibody were examined in all cases. Immunoreactive cells were presented with yellow brown staining in the cell. The positive cells were counted in 25 random fields from five cross-sections in each sample. The mean integral optical density (IOD) of positive cells was then determined.

*Colonic RNA and Fecal DNA Isolation and Quantitative RT-PCR Analysis.* MyD88, MUC2, Villin, Tff3 and GAPDH: Total RNA was purified using a reverse transcription kit (Thermo Fisher Scientific, Boston, USA). cDNAs were synthesized from RNA by reverse transcription of 2 μg total RNA. Oligo dT and RNase-free H2O were incubated at 65 °C for 10 min, placed on ice for 5 min, followed by the addition of 4 μl of 5 × buffer, 1.5 μl of a dNTP mixture, 1 μl of RNase inhibitor, and 1 μl of reverse transcriptase to reach a total reaction volume of 20 μL that was incubated at 42 °C for 60 min. cDNA was stored at -20 °C for the general chain reaction (PCR). The general PCR amplification system contained 2 μl of sample cDNA, 10 μl of GoTaq®Green Master Mix (M7122, Promega, USA), 0.2 μl of primers, and 7.2 μl of ddH2O. PCR was performed with an initial incubation step for 5 min at 95 °C, followed by 30–32 cycles of 95 °C for 30 s, annealing at 52 °C for 30 s, extension at 72 °C for 30 s, and extension for 5 min at 72 °C. Melting curve analysis was used to confirm the formation of the expected PCR products, and products from all assays were assessed via 1% agarose gel electrophoresis to confirm the correct lengths. The maximum OD value of the bands was analyzed using the Gel-Pro Analyzer 4.5 (Media Cybernetics, Rockville, MD, USA). The relative mRNA levels were normalized to the maximum OD value of GAPDH.

Firmicutes, Bacteroidetes, Proteobacteria, *Aeromonas* and *Escherichia coli*: The primer design software Primer Premier 5.0 is used to design the PCR primers of intestinal microbiota. And in the BLAST gene library (www.ncbi.nlm.nih.gov/BLAST), the corresponding genus specificity of the primer sequence is compared. Use the genomic DNA of the isolate as a template for PCR amplification. The reaction system (30 μL in total) is: 2 × Taq PCR Master Mix (Tiangen Biochemical Technology Beijing Co., Ltd.) 15 μL, template DNA 6 μL, ddH2O 7 μL, and primers 1 μL each. The reaction conditions are: 94 ℃ predenaturation for 5 min; 94 ℃ denaturation for 30 s, 55 ℃ annealing for 30 s, 72 ℃ extension for 90 s, a total of 35 cycles Ring; incubate at 72 ℃ for 10 min. The experiments were repeated three times.

*Determination of ROS Formation.* The suspension was loaded using DCFH-DA solution at a final concentration of 50 M and incubated for 30 min at 37 °C. Then, the samples were centrifuged at 1,000 rpm for 5 min (4 °C), and the cells were resuspended in phosphate-buffered saline (PBS, pH 7.2-7.4). For each treatment, 19105 cells were counted, and the experiment was performed in triplicate. Fluorescence was detected using a fluorescence microplate reader (excitation 488 nm and emission 525 nm).

*Western Blotting.* Total protein was extracted using lysis buffer (62.5 mmol/L Tris-HCl, 2% SDS, 10% glycerol; pH 6.8). After centrifugation at 12,000 × g for 10 min at 4 °C, the supernatants were collected. The protein concentration was determined using a bicinchoninic acid (BCA) kit (Beyotime, P0012). A sample of 20 µg of protein was electrophoresed using 10% sodium dodecyl sulphate-polyacrylamide gel electrophoresis. After electrotransferring the samples onto a polyvinylidene difluoride membrane (Millipore, Billerica, MA, USA), p-P65, p-IκB, and p-GSK-3β protein were blocked with 1% BSA and other proteins were blocked with 5% skim milk in 1 × Tris-buffered saline (TBS) with Tween (TBST) for 2 h at room temperature. The membranes were incubated with the monoclonal rabbit anti-mouse primary antibodies (GAPDH, 1:2000; MUC2, 1:1000; TLR4, 1:1000; p-GSK-3β, 1:1000; β-catenin, 1:1000; MT1, 1:1000; MT2, 1:1000; p-P65, 1:1000; p-IκB, 1:1000. Abcam, Cambridge, MA, USA) overnight at 4 °C. After washing with TBST, they were incubated with horseradish peroxidase-conjugated goat anti-rabbit IgG (1:5000, CW0103; CoWin Biotech Co., Inc.) for 2 hours at 37 °C. The immunoblots were performed using an eECL western blot kit (CW0049; CoWin Biotech Co., Inc.). The bands obtained in the blots were scanned and measured using ImageJ (version 4.0.2; Scion Corp., Frederick, MD, USA). Data are expressed as the integral optical density (IOD) of the bands, and the results were obtained from three repeated experiments.

**Supplementary Figures**

*3.1. Melatonin Improved SD-induced Aeromonas and LPS Increase and MUC2 Deficiency in Mice.*

*
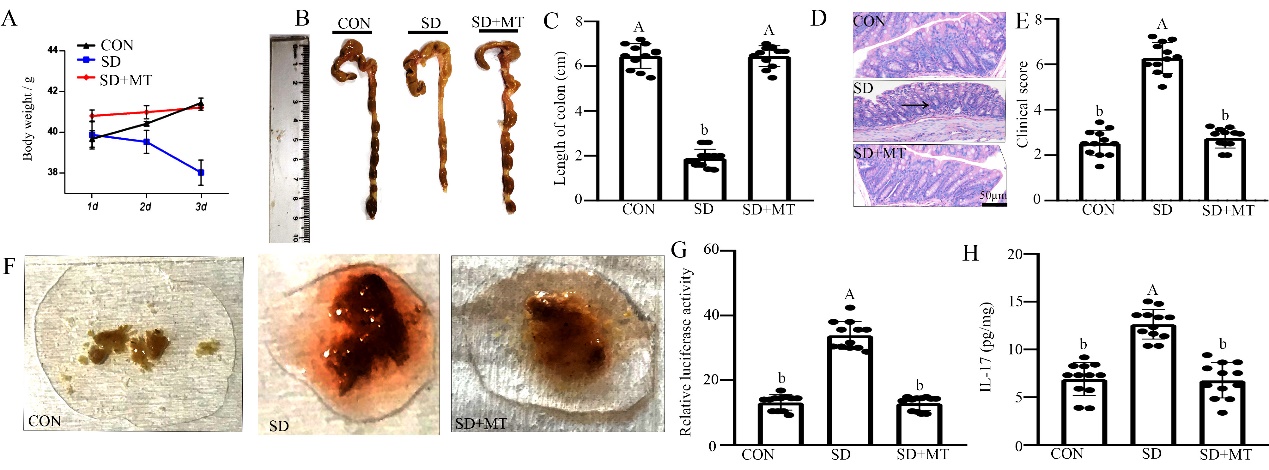
*

**Figure S1**. Melatonin improved SD induced colitis in mice. (A) Body weight; (B), (C) colonic length; (D) H&E staining photographs (scale: 50 µm); (E) histopathological score; (F) fecal occult blood; (G) relative luciferase activity for colonic permeability; (H) IL-17 concentrations in the colon of the CON, SD and SD+MT groups.

*3.2. FMT Promotes Reestablishment of the Intestinal Microecology.*

*
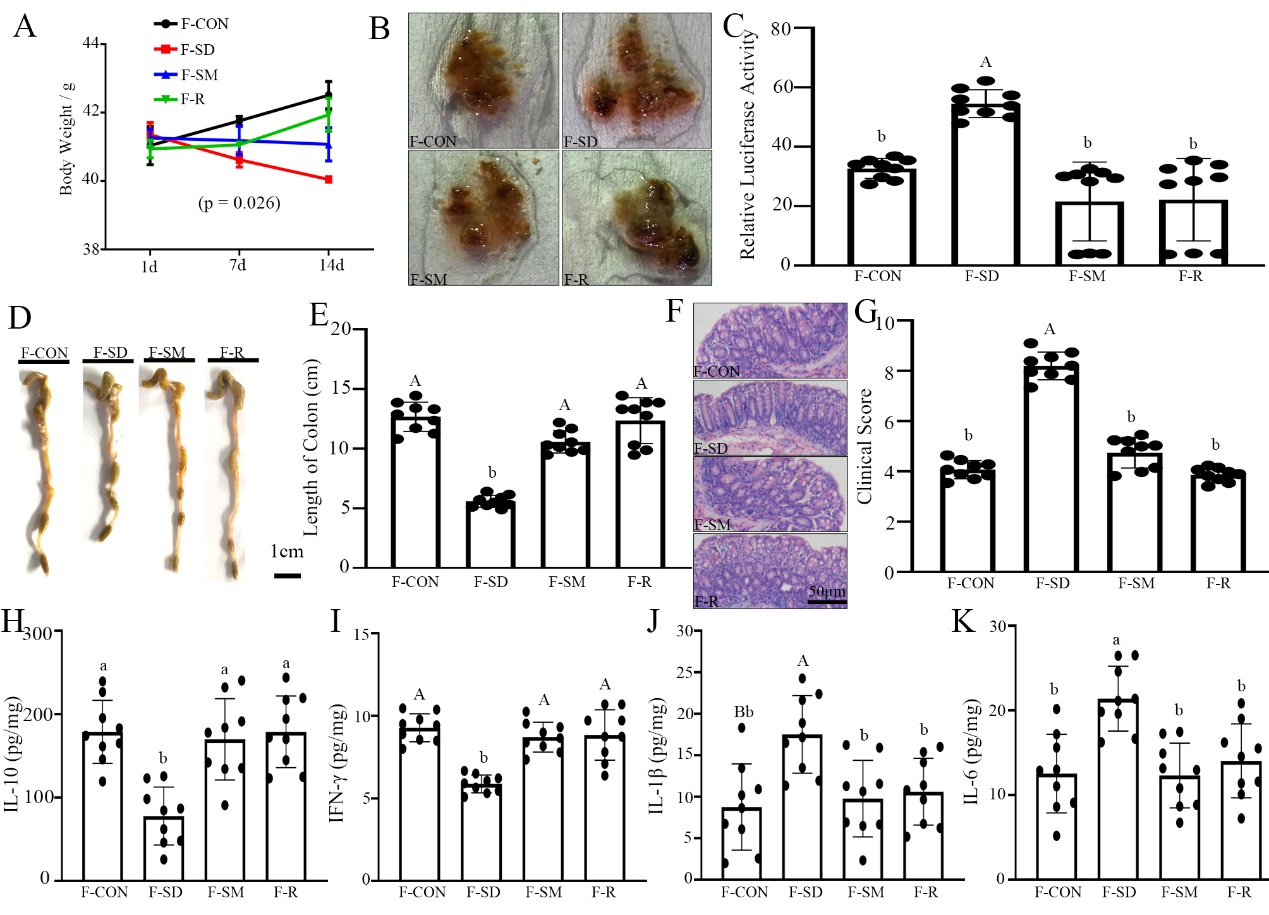
*

**Figure S2**. FMT re-established the intestinal microecology similar to CON, SD, and SD + MT mice. (A) Body weight; (B) fecal occult blood; (C) relative luciferase activity for colonic permeability; (D), (E) colonic length; (F) H&E staining photographs (scale: 50 µm); (G) histopathological score; (H–K): IL-10 (H), IFN-γ (I), IL-1β (J), IL-6 (K) concentrations in the colon of the F-CON, F-SD, F-SM, and F-R groups.


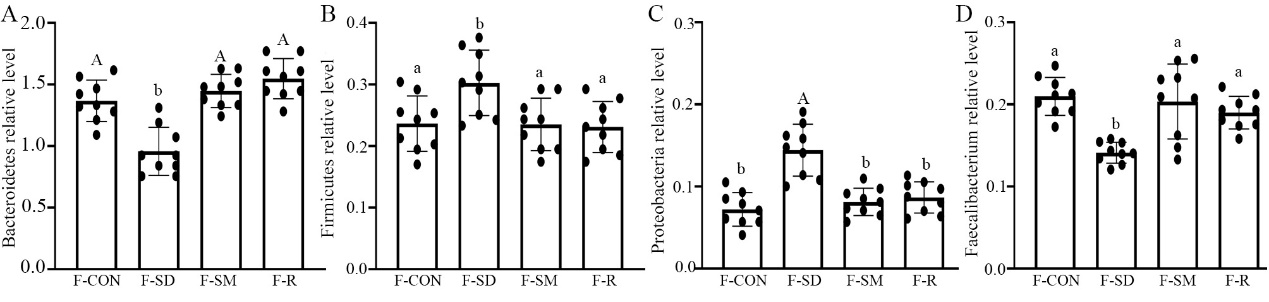


**Figure S3**. FMT re-established the intestinal microbiota similar to CON, SD, and SD + MT mice. The relative abundance of Bacteroidetes (A), Firmicutes (B), Proteobacterium (C) and Faecalibacterium (D) in F-CON, F-SD, F-SM and F-R group.

*3.3. A. veronii Colonization Promoted the Occurrence of Colitis and MUC2 Deficiency in Mice.*

**
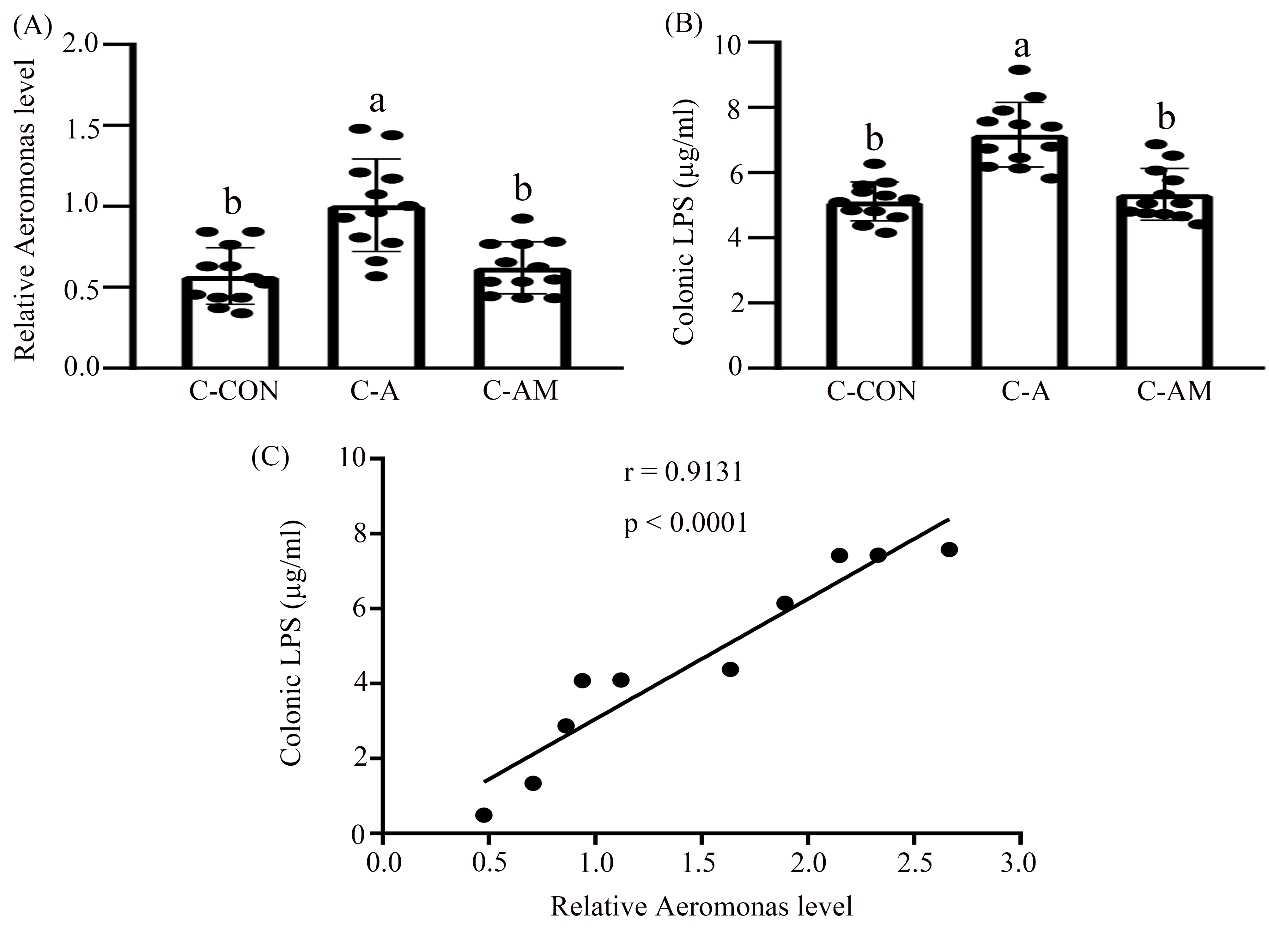
**

**Figure S4.** *Aeromonas* colonization elevated the relative abundance of *Aeromonas* and LPS in mice. Relative abundance of colonic (A) *Aeromonas* and (B) LPS in C-CON, C-A and C-AM groups; Correlation between *Aeromonas* level and LPS level (C).

*3.4. Melatonin Ameliorates LPS-induced Colitis.*

*
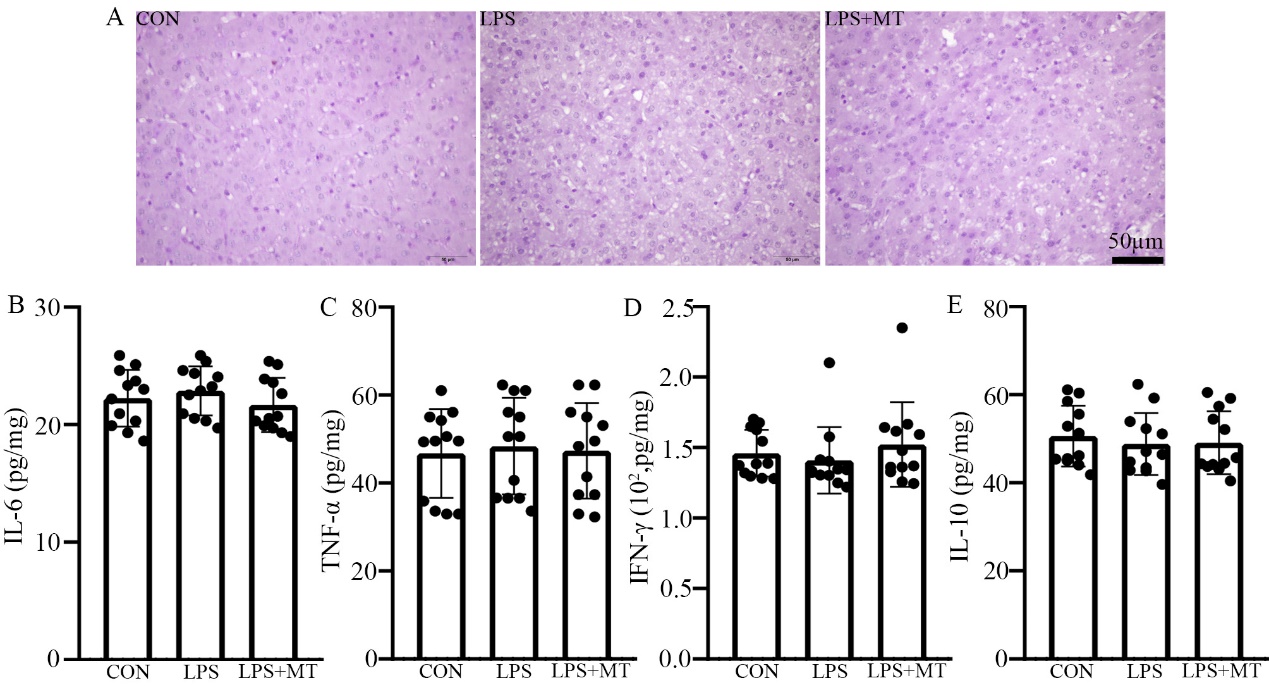
*

**Figure S5.** *Aeromonas* colonization had no effect on the liver. (A) H&E staining photographs of liver (scale: 50 µm); (B–E): IL-6 (B), TNF-α (C), IFN-γ (D), IL-10 (E) concentrations in the liver of the CON, LPS and LPS+MT groups.

*3.5. Melatonin Ameliorates LPS-induced MUC2 Depletion and Changes in the Expression Levels of Signalling Proteins in Mice.*

**
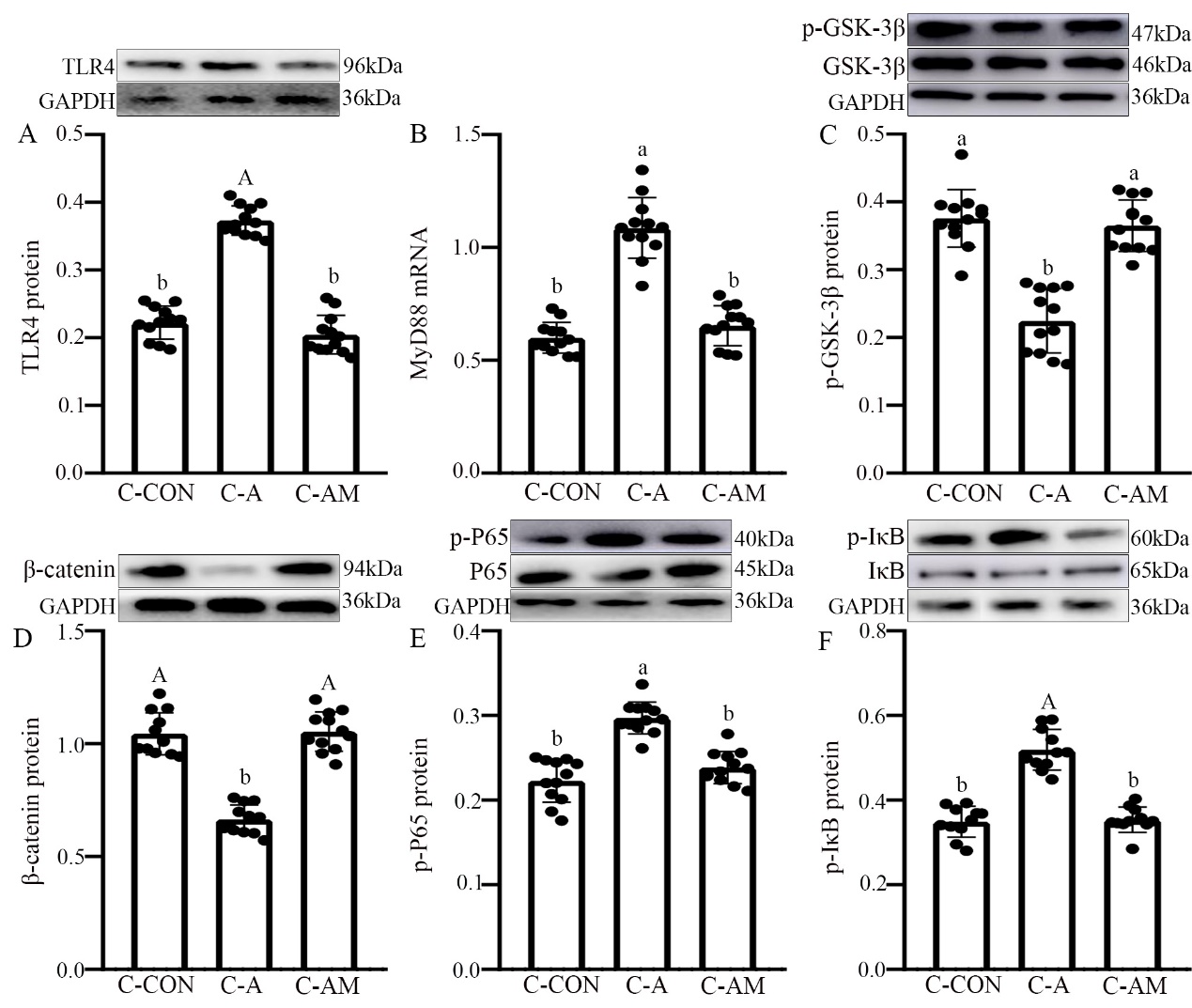
**

**Figure S6.** *Aeromonas* veronii colonization induced the changes of expression levels in signalling proteins in mice. (A) Colonic TLR4, (B) MyD88, (C) p-GSK-3β, (D) β-catenin, (E) p-P65 and (F) p-IκB proteins and mRNA production in the C-CON, C-A and C-AM groups.

*3.6. Melatonin Regulates the Level of MUC2 in HT-29 Cells Treated with A. veronii.*

**
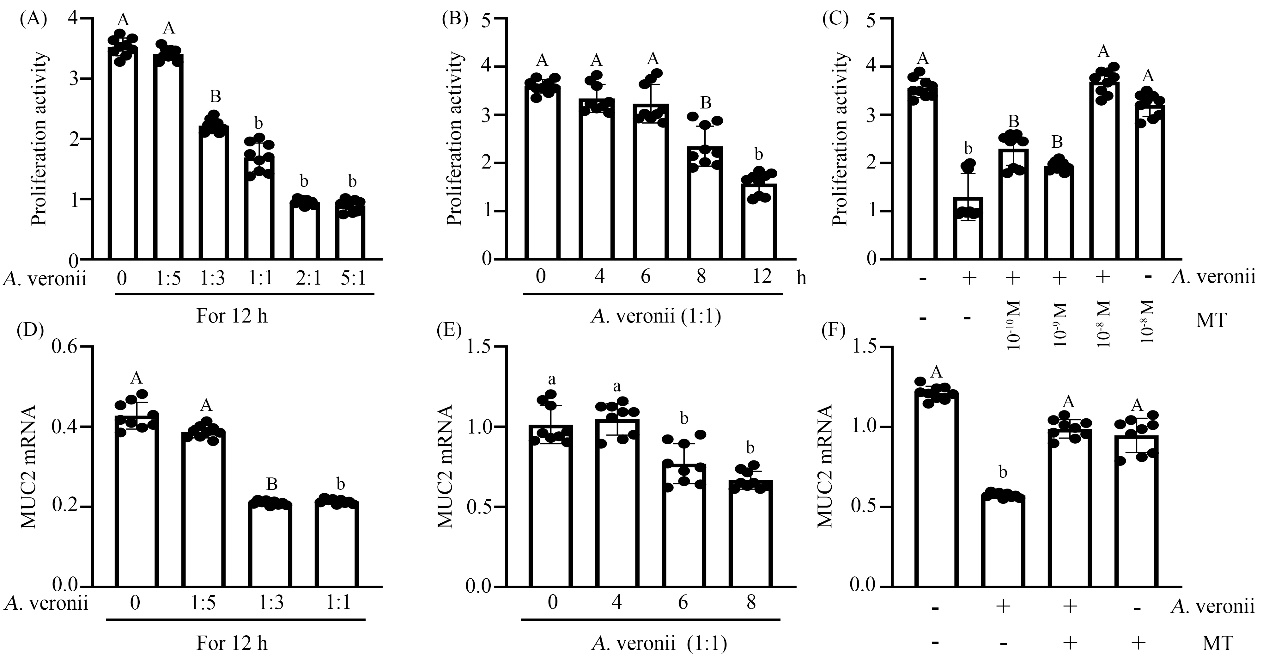
**

**Figure S7.** Melatonin improved *Aeromonas* induced MUC2 deficiency in HT-29 cells. (A) Proliferation activity in different proportion of *Aeromonas* (0-5:1), who were applied for 12 hours; (B) Proliferation activity in 1:1 *Aeromonas* colonization, who were applied for 0-12 hours; (C) Proliferation activity in different concentration of MT (0-10^8^M), exposed to *Aeromonas* supplementation; (D) MUC2 mRNA in different proportion of *Aeromonas* (0-5:1), who were applied for 12 hours; (E) MUC2 mRNA 1:1 *Aeromonas* colonization, who were applied for 0-8 hours and (F) MUC2 mRNA in various treatment groups.

*3.8. Melatonin Regulates the Level of MUC2 in HT-29 Cells Treated with LPS.*


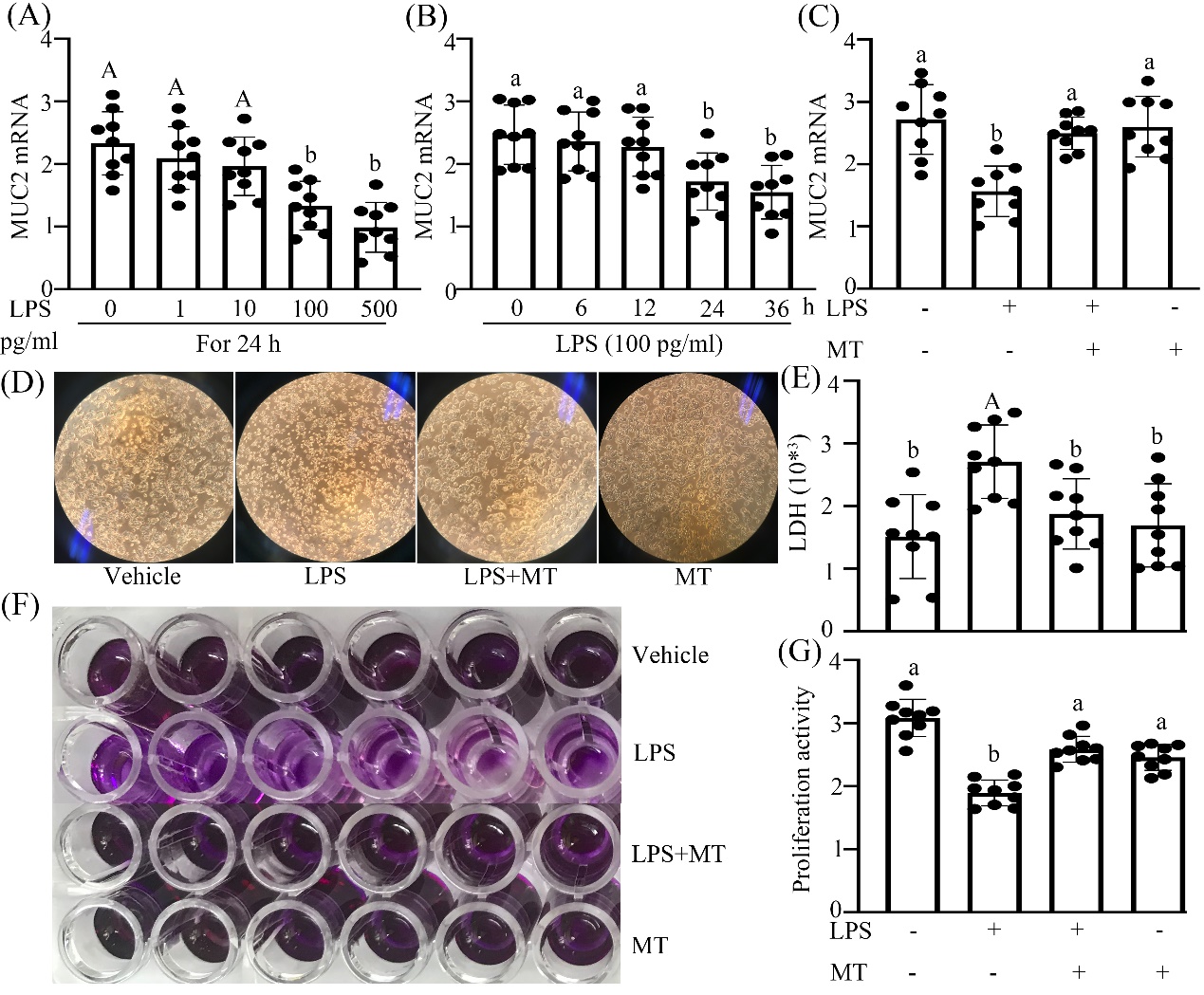


**Figure S8** Melatonin improved LPS induced MUC2 deficiency in HT-29 cells. (A) MUC2 mRNA in different concentrations of LPS (0-500 pg/ml), who were applied for 24 hours; (B) MUC2 mRNA in 100 pg/ml LPS, who were applied for 0-36 hours; MUC2 mRNA (C), cell morphology (D), LDH index (E), image of MTT assay (F) and cell proliferation activity (G) in various treatment groups.

**Graphical Abstract**


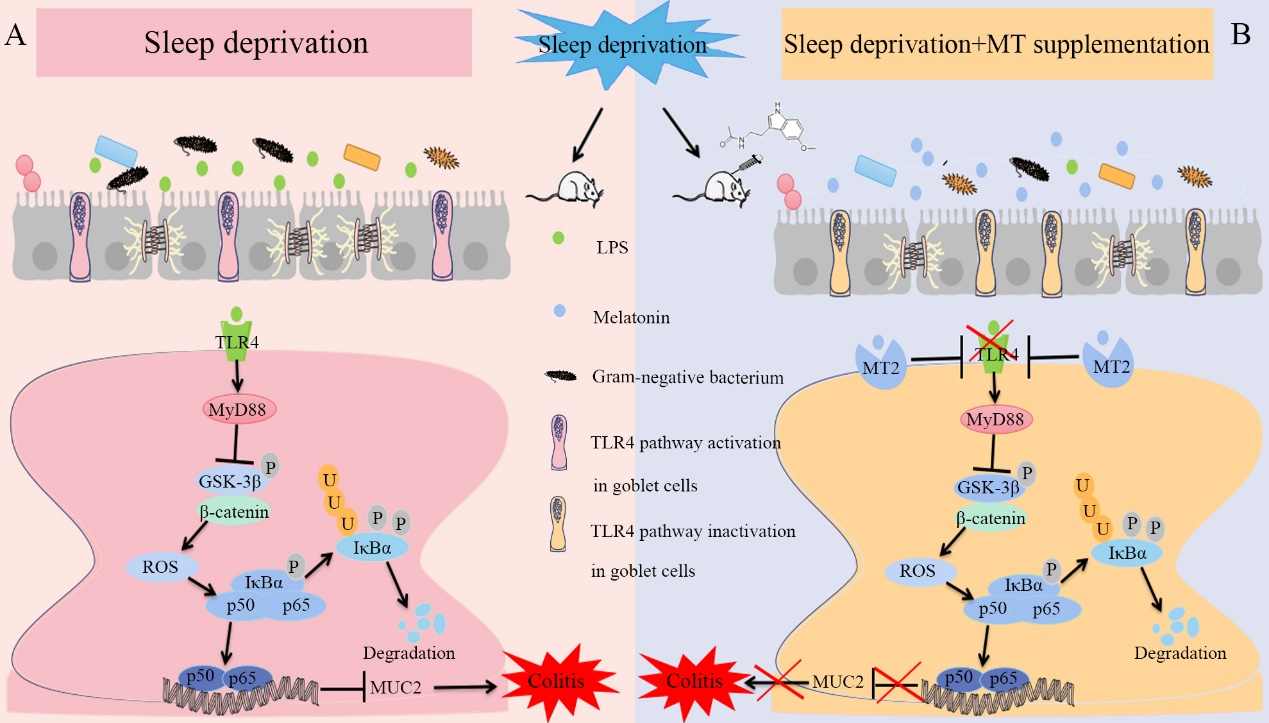

Supplement: Supplementary Materials — See supplementary methods and Figures S1-S8 in the Supplementary Material for comprehensive image analysis. [file 8133310.f1.docx]
